# Proteome-wide Structural Analysis of PTM Hotspots Reveals Regulatory Elements Predicted to Impact Biological Function and Disease*[Fn FN2]

**DOI:** 10.1074/mcp.M116.062331

**Published:** 2016-10-03

**Authors:** Matthew P. Torres, Henry Dewhurst, Niveda Sundararaman

**Affiliations:** From the School of Biological Sciences; Georgia Institute of Technology; Atlanta, Georgia 30332

## Abstract

Post-translational modifications (PTMs) regulate protein behavior through modulation of protein-protein interactions, enzymatic activity, and protein stability essential in the translation of genotype to phenotype in eukaryotes. Currently, less than 4% of all eukaryotic PTMs are reported to have biological function - a statistic that continues to decrease with an increasing rate of PTM detection. Previously, we developed SAPH-ire (*Structural Analysis of PTM Hotspots*) - a method for the prioritization of PTM function potential that has been used effectively to reveal novel PTM regulatory elements in discrete protein families (Dewhurst *et al.*, 2015). Here, we apply SAPH-ire to the set of eukaryotic protein families containing experimental PTM and 3D structure data - capturing 1,325 protein families with 50,839 unique PTM sites organized into 31,747 *modified alignment positions* (MAPs), of which 2010 (∼6%) possess known biological function. Here, we show that using an artificial neural network model (SAPH-ire NN) trained to identify MAP hotspots with biological function results in prediction outcomes that far surpass the use of single hotspot features, including nearest neighbor PTM clustering methods. We find the greatest enhancement in prediction for positions with PTM counts of five or less, which represent 98% of all MAPs in the eukaryotic proteome and 90% of all MAPs found to have biological function. Analysis of the top 1092 MAP hotspots revealed 267 of truly unknown function (containing 5443 distinct PTMs). Of these, 165 hotspots could be mapped to human KEGG pathways for normal and/or disease physiology. Many high-ranking hotspots were also found to be disease-associated pathogenic sites of amino acid substitution despite the lack of observable PTM in the human protein family member. Taken together, these experiments demonstrate that the functional relevance of a PTM can be predicted very effectively by neural network models, revealing a large but testable body of potential regulatory elements that impact hundreds of different biological processes important in eukaryotic biology and human health.

Since the discovery of phosphorylation in 1954 (1), post-translational modifications (PTMs)^1^ have emerged as a broad class of protein feature that expand the functional proteome in eukaryotes. Improvements in the detection of PTMs by mass spectrometry have resulted in an exponential increase in our knowledge of the number and type of PTMs that make up the landscape of a modified eukaryotic proteome. As a result, the rate at which PTMs are discovered now far surpasses the rate at which they can be experimentally tested for biological function - a characteristic that is specific for each PTM and likely not equivalent between all PTMs that have been observed (2–4). Thus, effective methods of prioritization are essential for quantifying the likelihood of a site to be regulatory and/or impactful on biological function, which we refer to as the *function potential* of a PTM.

Several unique features have been identified as predictors of biological impact for any given PTM - the determination of which relies on placing each PTM in the context of a multiple sequence alignment for a discrete protein or domain family, which we refer to as a Modified Alignment Position (MAP). For example, MAPs that are evolutionarily well conserved are more likely to exhibit biological function (3, 4). Similarly, functional PTMs are more commonly found within MAPs that exhibit a higher PTM observation frequency, are dynamic with respect to biological condition, located at protein interaction interfaces, and more solvent-accessible within a folded protein structure (5–7). Although efforts to elucidate the features associated with functional PTMs are relatively longstanding, few if any have established an integrative approach to quantitatively prioritize the function potential of PTMs beyond the use of single features.

Previous evidence from our lab first demonstrated that multiple feature integration can improve functional prioritization. To accomplish this, we built Structural Analysis of PTM Hotspots (SAPH-ire)—an algorithm through which multiple predictors of PTM function are integrated to produce a single, quantitative *function potential (FP*) score that rank orders each hotspot within or between protein families (6) (Fig. 1). Previously, we used SAPH-ire to predict novel PTM regulatory elements in G protein families—including heterotrimeric G proteins—for which we discovered and experimentally confirmed a novel PTM regulatory element that is critical for cell signaling (6, 8). We propose that similar analysis of PTMs across the entire eukaryotic proteome is likely to result in the discovery of several novel regulatory elements that have yet to be realized.

Here we apply SAPH-ire to protein families for which PTMs and protein structure are currently available, resulting in function potential prediction for 50,839 experimental PTM sites distributed across 31,747 MAPs. Using a neural network model (SAPH-ire NN) trained to predict the identity of embedded known-function MAPs, we derived a probability score that allows rank ordering for the likelihood of function for all MAPs including those with unknown function. We show that the SAPH-ire NN model significantly outperforms all other single or multi-feature predictive models and exhibits a proportional increase in predictive power for known function hotspots that have been more frequently studied (and therefore published). Using a strictly conservative probability threshold, we characterized the top-ranked 1092 MAPs corresponding to “*function potential hotspots,*” revealing 267 with truly unknown function - a striking fraction of which are also found mutated in human disease irrespective of whether the human protein, specifically, contains an observed PTM.

## EXPERIMENTAL PROCEDURES

### 

#### 

##### Assembly of the SAPH-ire Data Set

The SAPH-ire method is described in detail elsewhere and provided the foundation for studies conducted here (6). Briefly, an internal MySQL database (Oracle Corp.) was created to house all PTM, sequence and structural data utilized for this study, wherein UniProt identifiers (UIDs) associated with 213,022 experimentally observed PTM sites retrieved from dbPTM3 were stored (9). Putative PTM sites were excluded from the analysis. Each identifier was then cross-referenced with x-ray crystallographic protein structures harbored in the Protein Data Bank (PDB) (10). We limited our structural data to x-ray crystallography resolved molecules because of the higher resolution and ease of molecular coordinate segregation as compared with nuclear magnetic resonance (NMR) resolved structures. This aggregate dataset was then further reduced to eliminate redundancy, resulting in a final set of UIDs. The sequence of each UID was then clustered into families according to InterPro “family” and “superfamily” classifications utilizing the more inclusive groupings to maximize input PTM data. Multi-FASTA files were generated for each family and subsequently aligned using the MUSCLE algorithm under default parameters (11). For SAPH-ire analysis, each family requires a minimum of one PTM observation and one associated crystal structure. Individual family size was determined by available data with membership ranging from 2 to 360 proteins. The final input set was comprised of 1,325 families containing 13,267 unique proteins (from 1080 distinct eukaryotic organisms), for which there are 50,839 distinct PTMs coalesced into 31,747 MAPs.

##### Acquisition and Processing of Structural Inputs from the PDB

To meet the goal of including only high-quality, well-defined structures, we applied several filters to structural data obtained from the PDB. The PDB contains structures for many small peptide sequences which lack folding required for the tertiary and quaternary structures of the mature, functional proteins we aim to examine. Additionally, because of the lack of neighboring residues in three-dimensional space, the solvent accessibility for these residues are artificially inflated and can thereby impact family-relative ranking of PTM hotspots. We included only molecular structures with greater than 50 residues resolved by x-ray crystallography, which excluded only 5.3% of available crystal structures in the SAPH-ire data set. The distribution of resolved residues across all crystal structures in the PDB exhibits a natural trough at the 50 residue cutoff. In order to limit the impact of noncanonical sequences, we extracted the resolved sequence of amino acids from each PDB file and aligned each separately with the canonical sequence in UniProt. Any molecular structure with gaps in their aligned sequences—indicative of a noncanonical sequence—were excluded from this analysis. This filter effectively eliminates all chimeric and insertion sequences from the input set. We then used the POPS algorithm to calculate the solvent accessible surface area (SASA) for each computationally segregated chain (12). Consequently, structures that lack sufficient resolution for SASA calculation via POPS were excluded from the input data. The set of crystal structures used for analysis includes 49,685 unique molecular structures. To identify residues located at protein-protein interfaces (PPI), we cross-referenced each PTM with ProtInDB (13).

In several cases, regions of intrinsic structural disorder have been shown to act as regulatory elements and are often targets of modification (14–16). To account for the importance of these unresolved sites, we used measures of intrinsic disorder defined by the IUPred application (17). Specifically, in cases where a residue was structurally unresolved and is predicted to be intrinsically disordered, a maximal SASA value observed for the available structure is assigned as the effective SASA. In cases where the residue is not resolved in the crystal structure, but is predicted to adopt an ordered structure, no SASA value is incorporated from that specific protein/residue. Within the 31,747 MAPs analyzed here, 9428 occurred at positions of intrinsic disorder.

##### Definition of “Function Potential Hotspots” Identified by SAPH-ire

**Fig. 1.**
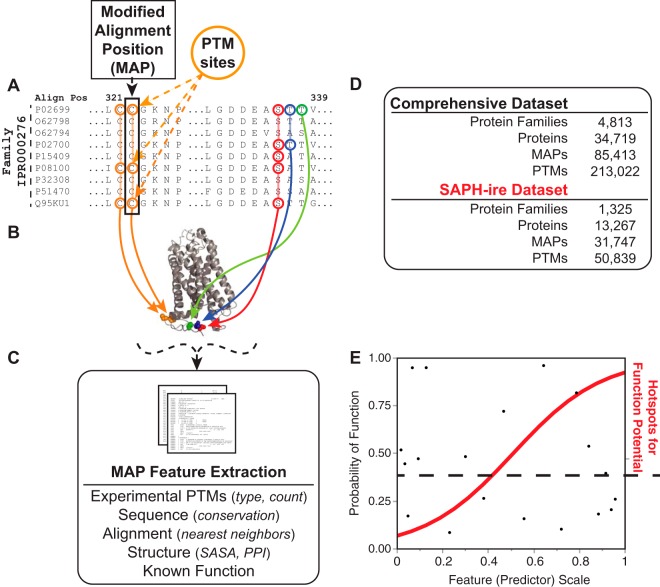
**Schematic diagram of SAPH-ire.** *A*, A theoretical segment of the multiple sequence alignment for a protein family (IPR000276; G protein-coupled receptor, rhodopsin-like) used here for illustrating the concept of SAPH-ire. *Circled* amino acid residues represent PTM sites experimentally observed on respective protein family members. *Circle and arrow color* represents the PTM observation frequency at each aligned position, called a MAP (modified alignment position), where *green* indicates 1 observation, *blue* for 2, *orange* for 3, and *red* for 5 or more. *B*, Cartoon rendering of bovine rhodopsin (P02699, RHO; PDB 2PED, chain A) showing side chains with projected PTM hotspots colored according to the number of observations within the family at each position aligned with the structural sequence. PDB coordinate data from the structurally projected PTM hotspots is used for calculation of solvent accessible surface area (SASA) and determination of protein interface residence (PPI). *C*, Hotspot features derived from the sequence and structural data are extracted for each protein family, where each hotspot corresponds to a precise family alignment position containing at least one PTM observation. *D*, Comparison of the comprehensive and SAPH-ire datasets representing all known experimental PTM data *versus* PTM data included in this study, respectively. *E*, Values calculated and derived from extracted hotspot features are analyzed by logistic regression or neural network models to produce probability scores for each hotspot.

MAPs with SAPH-ire NN probability scores of 0.196 or greater were labeled as “function potential hotspots”. This cutoff was derived from the frequency distribution of MAPs with known-function source counts of 11+ (KFSC ≥11). Hotspots with ≥35% probability score were surveyed for additional sources of known function as well as association with KEGG pathway maps. Importantly, our usage of the term “hotspot” is based entirely on the trained neural network model that integrates multiple MAP features—including those that harbor at least one biologically functional PTM site (Fig. 1*A*). This treatment is altogether different from previous descriptions of PTM hotspots that rely on identifying nonrandom spatial PTM clusters with respect to a single protein sequence and without reference to known-function PTMs (5, 18).

##### PTM Coincidence Analysis

MAPs for which more than one type of PTM has been observed were analyzed in isolation. Within this set, the number of coincidences between every possible pair of PTMs was tabulated as well as the number of observations and the relative percentage for each PTM in the entire coincident network. These values were organized into a network diagram of PTM coincidence using Cytoscape software (19), where each variable is plotted as edge thickness, node diameter, and node color, respectively. Frequency distributions for the coincidence count were then plotted with respect to the relative edge count. Coincident PTMs that are outliers in each distribution were highlighted using outlier box plots representing coincident PTMs that occur at a frequency greater than expected by the normal distribution (after normalizing for the frequency of node occurrences in the network). Relative edge count is calculated as ratio of the number of edges relative to the sum of the node occurrences (*i.e.* number of PTM observations; node diameter) in the coincident network scaled for ease of visualization. By using relative edge count, coincident PTMs can be compared directly without bias toward PTMs that are more frequently observed in general (*e.g.* phosphorylation and ubiquitination). A similar analysis was conducted to estimate the types of PTM coincidence that remain after zero-gap penalty alignment of the same protein families.

##### Logistic Regression, Neural Network Modeling, and Statistical Analysis

The comprehensive set of 31,747 MAPs in the SAPH-ire data set were analyzed and graphically displayed using JMP Pro 12 software (SAS, Inc.) and are available for download as an excel spreadsheet (supplemental data). The predictive power of individual features—PTM count, solvent accessible surface area (SASA), PTM residue conservation, protein-protein interface residence (PPI), neighbor count, neighboring known count—was estimated by nominal logistic regression analysis comparing feature values with respect to the functional state of each MAP. Any MAP harboring at least 1 biologically functional PTM (*i.e.* a *known-function* MAP) was designated “1,” whereas unknown-function MAPs were designated as “0.” To determine the predictive power, probabilistic models were analyzed by ROC curve analysis and AUC metrics. Chi-Squared hypothesis testing was used for AUC model comparisons. Neural network models were generated using a fully connected network architecture with a single hidden layer and hyperbolic tangent (*tanH*) transfer function wherein 33% of the data was subjected to random holdback for validation testing of the trained model. Individual predictors/inputs included PTM count, SASA, PTM residue conservation (described in (6)), PPI, number of neighboring MAPs within ± 2 alignment positions (*i.e.* neighbor count, NC), and number of neighboring MAPs with known biological function not including the position in question (a.k.a. neighbor known count, NKC). The comparison of model performance with respect to PTM count threshold was determined by excluding the data from positions beyond each threshold. Thus, a PTM count threshold of 3 corresponds to all MAPs with PTM count ranging from 1 to 3. Known-function source counts (KFSC) correspond to the number of literature references (curated by PhosphoSite Plus) that experimentally demonstrate biological functionality of the PTM. References were then filtered to exclude sources that lacked specific experimental evidence of functional impact (*e.g.* mass spectrometric PTM identification articles). The observed enrichment of MAPs above different scoring thresholds (0.1, 0.196, 0.35, 0.5) was also calculated with respect to enrichment expected by random chance (supplemental Fig. S4, S8).

##### KEGG Pathway and Human Gene Mutation Analysis

The complete list of UIDs for U4-type (truly unknown) unknown-function positions ≥ 35% probability were used to query the freely accessible KEGG human pathway database using KEGG mapper freeware (http://www.genome.jp/kegg/pathway.html) (20). The resulting pathway data was cross-referenced with the SAPH-ire dataset to evaluate function potential hotspots for each pathway.

Human disease-associated amino acid substitutions were collected from the publically available version of the Human Gene Mutation (HGMD) or NCBI ClinVar databases (21, 22). The UID, native position, amino acid substitution, and disease relationship of each mutation was related to the SAPH-ire data set through either UID native position (for Type-1 coincidence) or family alignment position (for Type-2 coincidence) to identify mutations overlapping with PTM sites or hotspots, respectively.

## RESULTS

### 

#### 

##### The SAPH-ire Data Set: PTMs and Protein Structure

**Fig. 2.**
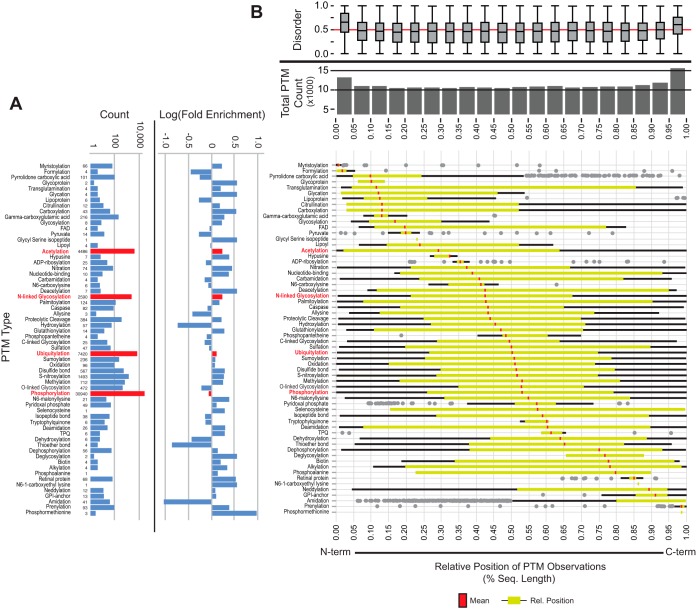
**The SAPH-ire data set.** *A*, Absolute count of PTMs observed by type (left). Log fold-enrichment of each PTM type in the SAPH-ire dataset corresponding to the ratio between percent representation in the SAPH-ire *versus* comprehensive datasets (right). 62/63 are shown; N6-succinyllysine not shown. *B*, Total PTM count and PTM site disorder tendency distributions relative to the location of experimentally identified PTM sites in the primary structure of all proteins in the comprehensive dataset (top). Disorder tendencies > 0.5 indicate structural disorder. PTM sites from the comprehensive dataset are organized by position relative to the size of the modified protein in amino acid residues. Distribution of each PTM type relative to the N-to-C termini of modified proteins (bottom). *Green outlier box plots* representing −25% to 75% portion of the distribution (median indicated by *red line*) with *black whiskers* representing minimum or maximum values corresponding to −25% or +75% ± [1.5 × interquartile range]. *Gray circles* represent outliers falling far outside the normal distribution.

As of the submission date of this manuscript, the collection of experimentally verified eukaryotic data available from dbPTM included 213,022 eukaryotic PTMs (referred to hear as the comprehensive PTM data set) that we coalesced into 85,443 MAPs distributed across 4813 protein families. Of these, 50,839 (∼24%) PTMs, 31,747 (∼37%) MAPs, and 1325 (∼28%) protein families can be analyzed by SAPH-ire, which requires experimental nonchimeric structures and experimental PTM data (the SAPH-ire data set) (Fig. 1*D*; see methods) (9). Within the SAPH-ire data set a total of 63 distinct types of PTM have been observed experimentally (Fig. 2*A*), with phosphorylation (61% of all data), ubiquitination (11%), acetylation (9%), and N-glycosylation (5%) representing the most abundant modifications. Although phosphorylation in the SAPH-ire dataset is slightly under-represented relative to the comprehensive PTM data set, sites of acetylation, N-glycosylation, and ubiquitylation are over-represented by as much as 85% (Fig. 2*A*).

We determined whether the distribution of specific PTM types across primary structure might reveal distinguishing characteristics by normalizing the position of each modification site relative to the total length of the modified protein (Fig. 2*B*). Within the comprehensive dataset, the N- and C-terminal ends of proteins harbor the greatest number of PTMs compared with residues that are internal, for which PTMs are uniformly distributed (Fig. 2*B*, top). PTM sites found at either terminus are also more frequently disordered, on average, compared with internal sites which exhibit evenly distributed disorder tendencies. For the most part, frequently observed PTMs are widely distributed across protein primary structure, with the exception of acetylation, which is more N-terminally distributed because of a high proportion of identified N-terminal acetylation sites (23, 24). Only a few PTMs that are frequently observed (>1000 observations) were also found to exhibit a narrow primary structure distribution, some of which can be explained by the nature of their functional role. For example, myristoylation, which is a cotranslational modification necessary for membrane anchoring, is observed almost exclusively at the N terminus, whereas prenylation, another membrane-anchoring modification that requires proteolytic cleavage at CAAX box motifs, exhibits an extreme C-terminal distribution. Additional high frequency PTMs that exhibit narrow primary structure distributions include Gamma-carboxyglutamic acid (Gla), which is almost exclusively found within the first 10–15% of the primary structure. Not surprisingly, Gla modifications are restricted to GLA domains, where the fold structure promotes interaction with vitamin K-dependent carboxylases (25, 26). Proper protein folding of the GLA domain requires the free N terminus, and thus, the domain and the modification are never found in other regions of the protein (27). Taken together, these results suggest that the positional distribution of most PTMs from N- to C terminus, with only a few exceptions, is largely uniform.

##### PTM Type Coincidence in a Single Alignment Position Identifies Coregulatory PTMs

**Fig. 3.**
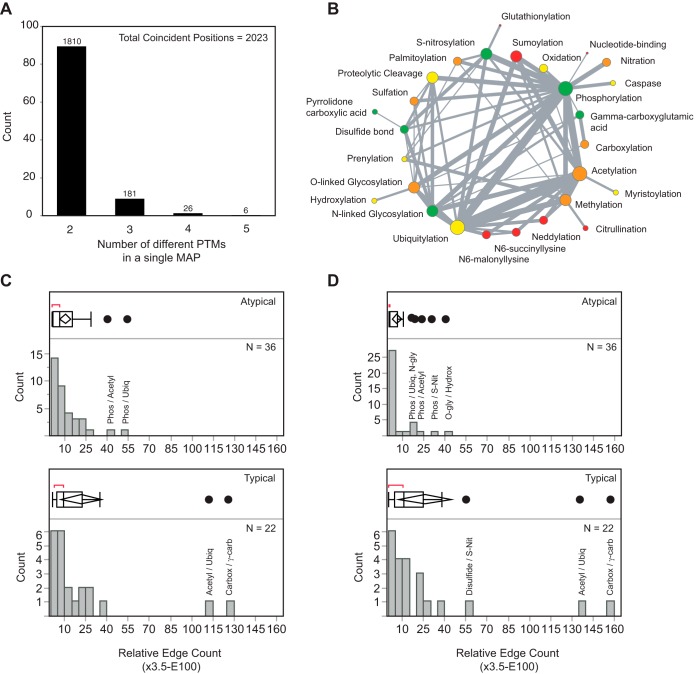
**Functional PTM coincidence may exist between modifications other than phosphorylation and ubiquitination.** *A*, The distribution of mixed-type PTM hotspots analyzed in the SAPH-ire dataset. *B*, PTM coincidence network for all coincident PTMs in the SAPH-ire dataset. Node size corresponds to the occurrence frequency of the PTM type in the coincident dataset. Node color corresponds to the percentage of occurrence for each PTM type in the coincident network relative to the occurrence in the total SAPH-ire dataset [*green* = <25%, *yellow* = 25–50%, *orange* = 50–75%, *red* = >75%]. Edge size indicates the number of coincident occurrences observed between two types of PTM. *C*, Identification of coincident PTMs that are outliers relative to the normal distribution of all coincident events. Normal distribution indicated by box plots based on standard multiple sequence alignment. *D*, Same as (*C*) but using zero-gap penalty multiple sequence alignment as described in text. *Atypical* = coincidence between PTMs that cannot modify the same residue. *Typical* = coincidence between PTMs that can modify the same residue.

Because of the PTM type-agnostic nature of SAPH-ire, the coincidence of different PTM types within a single MAP can be evaluated. PTM coincidence is of particular interest as it may reveal protein structures/regions that serve as evolutionarily conserved substrates for multiple types of enzyme. PTM coincidence may also reveal cases in which the identity of a regulatory PTM has shifted—representing PTM plasticity within a position. Within the SAPH-ire data set, we found that 2023 MAPs (6.6%) harbor more than one PTM type. Of these, 1810 (89.5%) contained 2 PTM types, 181 (8.9%) contained 3, 26 (1.3%) contained 4, and 6 (0.3%) contained 5 different PTM types (Fig. 3*A*). The breadth of coincident observations was visualized in a coincidence network reflecting the relative frequency of pairwise coincidence for each participating PTM (Fig. 3*B*). More than half of all nodes in the coincident network (14 out of 27) correspond to PTM types that are more frequently coincident with other types of PTM based on their occurrence in the total SAPH-ire data set (Fig. 3*B*, orange and red nodes). Coincidence within a MAP is most commonly observed for the PTMs of lysine and arginine—sumoylation, neddylation, and methylation on lysine, and methylation and citrulination on arginine (Fig. 3*B*).

The coincidence of two different PTM types may be explained by a multitude of possibilities, which we analyzed in turn. Naturally, coincidence is more common for residues that can undergo different types of PTM (*typical* coincidence), such as ubiquitylation, acetylation, and sumoylation of lysine, which explains 23/63 (36.5%) of all edges in the network (Fig. 3*C*). Comparatively, the remaining 40 *atypical* coincident edges are lower in overall average frequency, but also reveal outliers of interest, including phosphorylation/ubiquitination and phosphorylation/acetylation edges (Fig. 3*C*). Consistent with this observation, phosphorylation, ubiquitylation, and acetylation have been shown previously to be cooperative and coregulatory PTMs that control protein fate *in vivo* (5, 28, 29). Additional atypical coincident PTMs include phosphorylation/S-nitrosylation as well as N-glycosylation/proteolytic cleavage, phosphorylation, or disulfide bond formation. Thus, phosphorylation/ubiquitination and phosphorylation/acetylation coincidence, although abnormally abundant, are not the only forms of abundant PTM coincidence in eukaryotes.

We also evaluated PTM coincidence in the context of residue solvent accessibility and intrinsic disorder to determine if increased accessibility or disorder correlates with greater coincidence. To answer this question, we analyzed the frequency distribution of disorder tendency for the modified residues contained within each coincident position, corresponding to 6510 nonredundant disorder tendency values captured from 2023 coincident MAPs. The vast majority of the PTMs found in coincident MAPs (∼70%) occur on structurally ordered residues, consistent with the proportion of residues that are spatially resolved in crystal structures for the coincident and SAPH-ire datasets (supplemental Fig. S1*A*, *total*). Coincident positions harboring two or three different types of PTM reflect similar distributions of intrinsic disorder tendency. However, this trend reverses for positions harboring four and five different types of PTM, which represent 6% (396/6,510) of all coincident PTMs (supplemental Fig. S1*A*). We conclude that solvent accessibility and intrinsic disorder, alone cannot explain the majority of coincident PTM types observed in the SAPH-ire dataset. Furthermore, positions in which coincidence exceeds 3 PTM types are extraordinarily rare and occur predominantly at sites that are intrinsically disordered.

Intrinsic disorder is common at the N- and C-terminal ends of proteins, regions that are also often difficult to align by sequence. To evaluate whether atypical PTM type coincidence was the result of multiple sequence alignment as opposed to evolutionary PTM plasticity, we performed zero gap-penalty multiple sequence alignment with just those families containing coincident PTMs. Eliminating the alignment gap penalty ensures that residue alignment is maximized at the cost of accumulating inserted gaps in the alignment sequence. Such alignments would largely disfavor the occurrence of coincident PTMs unless they were tightly constrained by neighboring residues, and in these cases would suggest that coincidence is not simply because of strict sequence alignment constraints. Surprisingly, we found an expanded set of atypical PTM coincidence outlying the normal distribution (Fig. 3*D*). These include phosphorylation and N-glycosylation or S-nitrosylation, as well as O-linked glycosylation and hydroxylation. Of these, all forms of atypical, zero gap-penalty coincidence except phosphorylation/acetylation and O-glycosylation/hydroxylation occur predominantly at sites that are structurally ordered and cannot be explained by an overabundant occurrence at sites of intrinsic disorder (supplemental Fig. S1*B*). Manual inspection of the zero gap-penalty alignments for each set of coincident PTM types revealed that many could be explained by the nature of the alignment and/or the PTMs observed within the family. For example, in zero gap-penalty alignments, a subset of more closely related members might harbor the observed PTM site whereas the remaining members have lost the PTM site altogether. Only a few cases could be found in which the sequence alignment could not provide an adequate explanation of coincidence (data not shown). Under these few circumstances, the retention of coincidence in a zero gap-penalty alignment suggests that cooperative coregulatory mechanisms aside from phosphorylation and ubiquitylation or phosphorylation and acetylation may also exist. We conclude that the coincidence of different PTM types within a single alignment position is reflective of coregulatory function. Furthermore, PTM plasticity as reflected by atypical PTM coincidence is rare within the subset of protein families that can be linked to a crystal structure.

##### The SAPH-ire Neural Network Model is a Robust Predictor of Functional PTM Hotspots

As described above, several unique MAP features are moderately predictive of function. To quantify the degree to which individual MAP features are predictive, we established logistic regression models with two nominal outcome classes: known and unknown function. In the SAPH-ire data set, 2010 MAPs harbor at least one PTM that has been experimentally demonstrated to have an impact on a biological process/protein function based on the PhosphoSite Plus database of PTM function (30). In contrast, 29,737 positions with unknown function correspond to any MAP that is not associated with sites contained in PhosphoSite Plus.

**Fig. 4.**
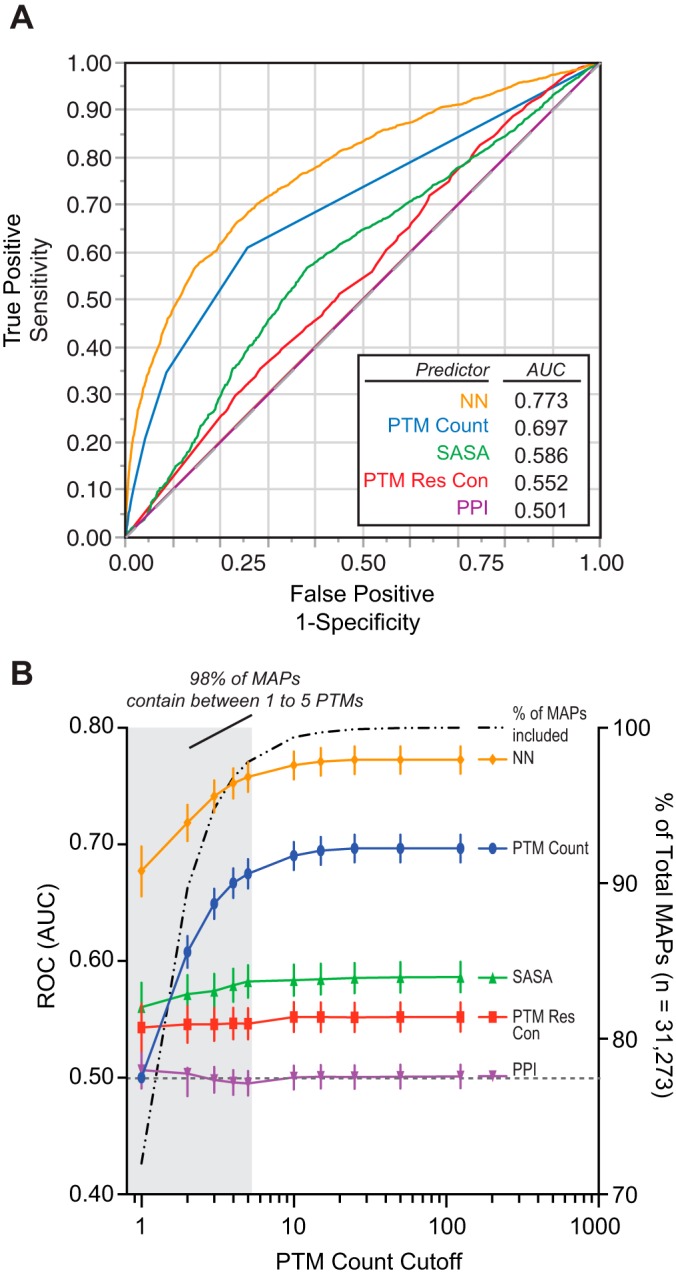
**SAPH-ire neural network model is a robust predictor of PTM function potential.** *A*, Representative ROC curves generated from single-predictor nominal logistic regression models or a neural network model based on multiple predictors. *B*, Analysis of prediction strength (as estimated by AUC analysis of ROC curves) relative to the PTM count threshold (described under Experimental Procedures). *Gray dashed lines* in *A* and *B* represent AUC = 0.5, which illustrates the expected ROC curve for a model that performs no better than random.

ROC curves and AUC metrics are ideal for evaluating the quality of different predictive models in cases where output probabilities need not be calibrated for outright prediction of class membership, where the classes are highly imbalanced (*i.e.* high number of one class *versus* another), and where rank ordering is the desired output (31). Consequently, ROC-AUC metrics can be used to estimate how effective a classifier (*i.e.* MAP feature) separates the outcome classes—known *versus* unknown function in this case. Good predictive models exhibit large AUC values (0.75 to 1.00) corresponding to higher rates of true positive compared with false positive prediction, whereas poor predictive models exhibit AUC values close to 0.5, corresponding to predictive power that is no better than random selection of known and unknown classes. Consistent with our previous results, we found that residue SASA and neighboring known count exhibit moderate predictive power for biological function (Fig. 4*A*; supplemental Fig. S2) (6). In comparison, the logistic regression model based on neighbor count, PTM residue conservation, and PPI is only slightly better than would be expected by random chance, whereas the model based on PTM count is a significantly more effective predictor, as illustrated by ROC-AUC analysis (Fig. 4*A*, supplemental Fig. S2).

Next, we compared single-feature logistic regression models with an artificial neural network (NN) model that integrates multiple hotspot features. Artificial neural networks utilize a machine-learning approach that effectively enables the integration of multiple regression models, each of which is transformed by a scaling factor (weight), that is optimized through iterative computation to maximize the correct assignment of the classes (*i.e.* assigning high probability to known-function MAPs) and minimize penalties associated with false prediction (32). We utilized a hyperbolic tangent (tanH) neural network model for prediction of MAPs with known function, whereby a randomly selected set of positions corresponding to 33% of the data set was held back as a validation dataset (see methods). Screening through several different network structures and combinations of input features, we settled on a fully connected neural network model with 6 feature variables feeding a single hidden layer containing 3 nodes (supplemental Fig. S2). Input features included PTM count, SASA, PTM residue conservation, PPI, count of neighboring positions (neighbor count) within a ± 2-residue window, and count of neighboring known-function positions within a ± 2-residue window. In contrast to single-feature regression models, the neural network model significantly outperformed all other models tested, including PTM count (AUC_PC_ = 0.697 *versus* AUC_ANN_ = 0.773, *X*^2^ = 218.97; *p* ≪ 2E-11) as well as the rationally-derived model (RD) (AUC_RD_ = 0.653 *versus* AUC_ANN_ =0.773, *X*^2^ = 417.89; *p* ≪ 2E-11), (Fig. 4*A*, supplemental Fig. S2).

Although PTM count is the most effective single predictive feature, it also exhibits a wide range of values across MAPs with known function. Therefore, we evaluated each model across a range of PTM counts. Logistic regression models that are based on PTM count alone will necessarily fail to correctly classify positions with PTM count = 1, which is problematic considering that 782 positions with known function (or ∼39% of all known-function MAPs in the SAPH-ire dataset) have an observed PTM count of 1. Moreover, the vast majority (>98%) of all MAPs (known or unknown) contain 5 or fewer PTM observations. Indeed, we found the PC logistic regression model performs progressively worse as a predictor of biological function for MAPs containing less than six PTMs, and is no better than random chance for hotspots with a single PTM (Fig. 4*B*). In contrast, the SAPH-ire NN model, which relies on the integration of several MAP features, improves the function potential prediction for MAPs, including those with low PTM count (AUC = 0.67). Thus, PTM count alone is ineffective as a predictor of biological function for the vast majority of MAPs. We conclude that the SAPH-ire NN model is a robust predictor of known-functional PTM hotspots.

##### The Predictive Power of the SAPH-ire Neural Network Model Increases Proportionally With the Confidence in Known Biological Function

MAPs for which there is experimental evidence of biological function are classified as “known” with as few as one source of published evidence. In the models developed here, we classified MAPs as either having known function (known function = 1) or unknown function (known function = 0), without including evidence of source count. In many cases, PTMs with well-established functional effects contain several sources of reference that independently corroborate the function experimentally. For example, several publications demonstrate the impact of lysine acetylation sites in histone proteins (33), or specific sites of phosphorylation in the tumor suppressor p53 (34), or activating phosphorylation of mitogen-activated protein kinases (35). Restated, PTMs with a high number of experimental sources result from the scientific community repeatedly confirming the function of a particular PTM, thereby increasing confidence in its classification as functional. Thus, the source count of known-function MAPs is proportional to the confidence in their classification as biologically functional.

**Fig. 5.**
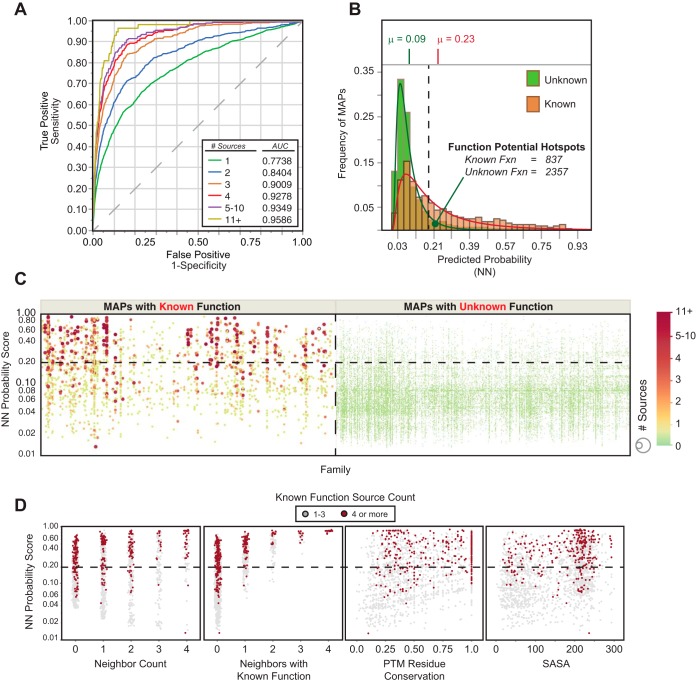
**The prediction strength of SAPH-ire NN improves with increasing known-function source count.** *A*, ROC-AUC analysis of logistic regressions for SAPH-ire NN probability scores across groups of variable known-function source counts. *B*, Overlaid frequency distributions for the entire population of known-function and unknown-function MAPs relative to the probability of function predicted by SAPH-ire NN (*x* axis). *Dashed line* delineates the cutoff for identifying function potential hotspots (at 0.196). *C*, Visual representation of 31,747 MAPs (*circles*) with known and unknown function for each family (*x* axis) where known-function source count is represented by color and size of the circle. Each circle corresponds to a unique MAP (modified alignment position) harboring experimental evidence of PTM from multiple members within a protein family. *Dashed line box* represents the function potential hotspot cutoff shown in *B. D*, Distribution of known-function MAPs with 4+ sources supporting biological function (KFSC 4+, *red circles*) relative to MAP neighbor count, neighboring known-function MAP count, PTM residue conservation, and SASA. *Grey circles* correspond to all other known-function MAPs.

We compared the count of unique sources for each known-function MAP within the SAPH-ire data set. Source counts for known function range between 1 and 68 unique references (supplemental data), which we grouped into six bins (KFSC 1, 2, 3, 4, 5–10, and 11+). We evaluated source count as a response relative to SAPH-ire NN probability scores using ordinal logistic fit models and ROC-AUC analysis (Fig. 5*A*). Known-function MAPs with a single source count were predicted very effectively (AUC_SC = 1_ = 0.774). Analysis of known-function MAPs with higher source count showed a continuous increase in ROC-AUC metric, reaching greater than 0.9 for hotspots with as few as 3 sources of supporting literature (Fig. 5*A*).

We conservatively defined function potential hotspots as MAPs with probability scores of 0.196, which we defined by the probability score above which 90% of all MAPs with KFSC ≥11 are included (supplemental Fig. S3). Using this strict cutoff, we identify 3230 function potential hotspots, 2357 of which have unknown function (Fig. 5*B* and 5*C*). Plotting each MAP with respect to protein family and probability score further shows that the increasing probability score for known function high source-count MAPs is largely family independent (Fig. 5*C*). Moreover, by comparing the enrichment observed *versus* expected by random chance occurrence at each KFSC level, we find that the NN model performs between 5 and 340 times better than would be expected by random chance alone (supplemental Fig. S4*A*, S8*B*). Because known function source count was not included as a feature during model generation, these data demonstrate that the SAPH-ire NN model is effective for identifying functional MAPs and predicting the degree to which they may have a biological impact.

Previous reports have described PTM hotspots in domain families as regions that are densely modified above what would be expected by random distribution of PTMs across the length of a given protein sequence (5, 18). In principle, such methods are intended to identify nonrandom PTM neighbors along the length of a protein sequence with the underlying hypothesis that highly enriched clusters are more likely to correspond to functional hotspots. Although altogether different from SAPH-ire, each method relies to some degree on the relationship of a given PTM site or MAP to its neighboring sites or positions. To evaluate the dependence of SAPH-ire NN output probabilities on nearest neighboring MAPs, we evaluated the distribution of individual known-function MAPs with respect to input features and probability score. As expected, the ranking of MAPs by SAPH-ire NN, although benefiting from inclusion of several features, is largely independent of any one feature (Fig. 5*D*, supplemental Fig. S4*B*). Furthermore, known-function MAPs exhibit a broad range and combination of feature values, not one of which is a perfect predictor for PTM biological function. Thus, prediction of PTM hotspot function potential requires consideration of several features for a given PTM site, which can be elucidated by neural network models.

##### Function Potential Hotspots are Found in Several Pathophysiological Human Pathways and Overlap With Disease-linked Mutations

**Fig. 6.**
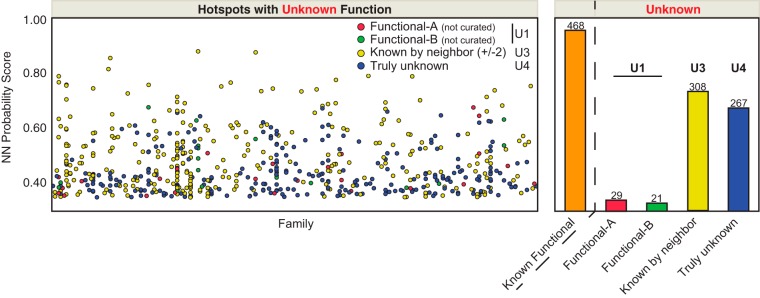
**Analysis of unknown-function hotspots with high function potential.** Function potential hotspots above 35% probability score were color-coded by category (see supplemental Fig. S5). Hotspots discovered to have biological function but not curated in the PSP database (U1-Functional-A); hotspots likely to have biological function but lacking complete evidence (U1-Functional-B); hotspots that are neighboring a known-function hotspot (U3); truly unknown-function hotspots that have yet to be studied (U4). Individual hotspots organized by family (*x* axis) (*left*). Bar graph showing quantification of each hotspot category observed with probability score > 35% (*right*).

We conducted a detailed analysis of the highest ranking function potential hotspots above 35% (1092 total MAPs), of which 468 and 624 were originally classified (during NN model development) as having known and unknown function, respectively (supplemental Fig. S5*A*). Of particular interest within this subset are the hotspots of unknown function, comprised of 5443 unique PTMs. Function potential hotspots with unknown-function are comprised of multiple sub-classes of PTM that were cryptic during SAPH-ire NN model development (see methods). Therefore, we classified each hotspot that was originally defined as unknown (by the PhosphoSite Plus database). Restated, these hotspots were labeled as having unknown function during NN model development (supplemental Fig. S5*B*). The categories include: (*U1*) hotspots that are biologically functional or likely functional based on published experimental evidence, but had not yet been curated in the PhosphoSite Plus database at the time of this study; (*U2*) hotspots that have been reported as having no biological function (a rare circumstance given that negative results are often left unreported and virtually impossible to prove outright); (*U3*) hotspots whose function has not yet been tested experimentally but can be assumed to be functional based on proximity to nearby known-function hotspots from the PhosphoSite Plus database; and (*U4*) hotspots whose functional impact is truly unknown. Using this classification scheme, we organized each of the 624 unknown-function hotspots into one of the 4 classes. Using manual literature searching methods, we identified 50 U1-type hotspots that are known to have function but were not contained within the public version of the PSP database at the time of this publication (Fig. 6). We split the U1 class into two groups: Function-A and Function-B, to delineate between PTMs with very clear evidence of biological function and PTMs for which the evidence for function was consequential rather than direct, respectively. We also included as known those PTMs that could be catalyzed by specific enzymes. Three hundred eight hotspots were proximal to a known function hotspot (U3), which we refer to as “known-by-neighbor.” The remaining 267 (43%) unknown-function hotspots were labeled as truly unknown (U4), and correspond to PTMs that have yet to be studied for functional impact, are located in a structural region that has not been investigated for PTM-dependent regulation, but have a high probability of being functionally important based on SAPH-ire NN predictions.

**Fig. 7.**
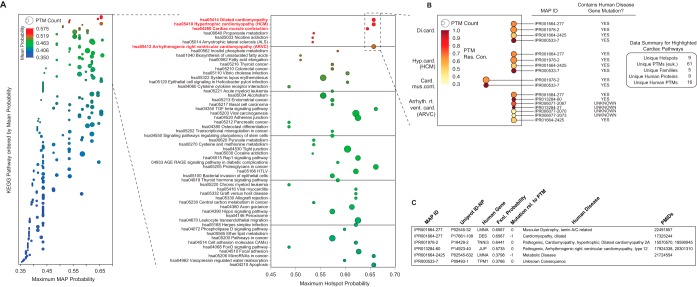
**Function-potential hotspots that are highly ranked by SAPH-ire NN impact several pathophysiological human pathways.** (*A*, *left*) Scatterplot showing maximum and mean probability of Kegg-pathway-specific hotspots (see also supplemental Fig. S6, S7). (*A*, *right*) Zoomed view of (*A*) with pathway labels. *B*, Deconvoution of PTM hotspot data for high-ranking KEGG pathways involved in cardiac function (see *dashed box* in *A*). *C*, HGMD and Clinvar analysis of cardiac-pathway hotspots that lack known function for PTMs in the hotspot with PubMed references describing each disease-associated mutation.

To investigate the breadth of biological functions covered by the U4-type hotspots above 0.35 probability score, we deconvoluted each hotspot into its component proteins, designated by UniProt Identifiers (UID) (supplemental data). The list of UIDs was used to query the human KEGG pathway database (20), resulting in the association of 211 human proteins from 165 hotspots comprised of 873 PTMs (PTM count; not all human) (supplemental Fig. S6). In order to highlight pathways enriched in hotspots with high function potential that are also frequently observed, we organized each pathway in terms of mean and maximum hotspot probability as well as PTM count (Fig. 7*A* and supplemental Fig. S7). Surprisingly, four highly enriched pathways shared involvement in cardiac function and cardiomyopathy (hsa04260, hsa05414, hsa05410, and hsa05412) (Fig. 7*A*). Within these four pathways, we observed 9 unique hotspots from 5 protein families containing 61 experimentally observed eukaryotic PTMs, 16 of which have been specifically observed on the human protein involved in the pathway (as opposed to another protein family member) (Fig. 7*B*).

In the absence of experimental evidence for PTM functionality in these cases, we opted to survey each of the nine pathway-specific hotspots/proteins for evidence of mutation in human disease. We queried the publically available version of the Human Gene Mutation Database (HGMD) as well as the Clinvar database available through NCBI using the UID-PTM site data from each hotspot (21, 22). We found that six out of the nine human proteins and five out of nine hotspots were associated with substitution mutations that are either causal or consequential with a variety of cardiac and other human diseases (22, 36–42). In all but two cases, the mutation was found to occur precisely at the PTM site and in the remaining two cases, is located at the −1 position relative to the hotspot (Fig. 7*C*). Taken together, these results suggested that function potential hotspots predicted by SAPH-ire NN to be biologically important for protein function, might also be important in disease etiology.

##### Human Disease-Linked Pathogenic Mutations Are Enriched In Function Potential Hotspots Identified By SAPH-ire NN

**Fig. 8.**
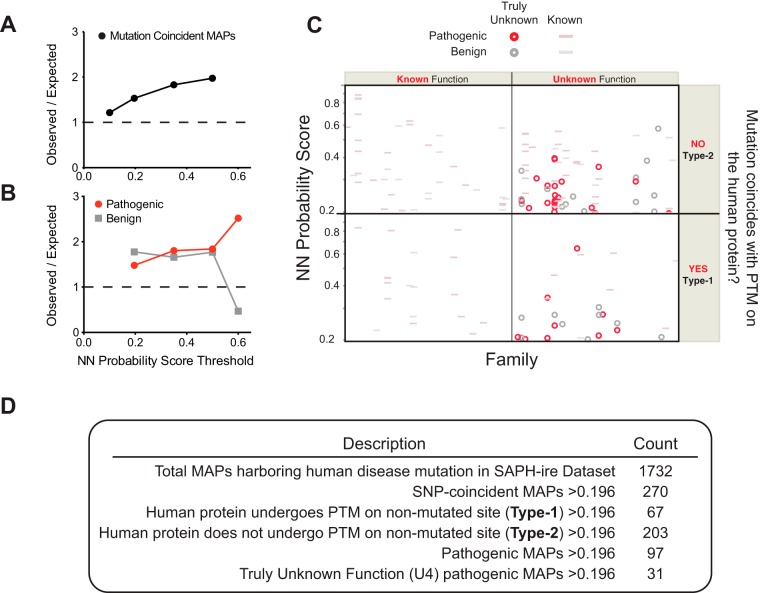
**Disease-linked pathogenic, but not benign mutations are enriched proportionally with SAPH-ire NN probability score.** *A*, Plot of observed frequency of SNP-coincident MAPs found above a given threshold relative to the frequency expected by chance. Dashed line represents ratio expected if no enrichment was observed. *B*, Same as in *A*, but specific to mutations that are pathogenic or benign. *C*, Scatterplot of SNP-coincident pathogenic or benign hotspots above 0.196 probability score. Truly unknown (U4) hotspots harboring pathogenic or benign mutations are highlighted whereas hotspots with known function or known by proximity are dimmed. *D*, Table showing the hotspot count for categories illustrated in *A*.

To further investigate the coincidence of disease-linked human mutations and function potential hotspots, we joined the comprehensive set of ClinVar substitution-causing SNPs to the SAPH-ire data set (supplemental data). Out of the 31,747 MAPs and 24,951 mutations, we found 1732 could be matched based on shared alignment position (supplemental Fig. S8*A*). We next evaluated the enrichment of SNP-coincident MAPs relative to the expected frequency by chance observation at different thresholds of SAPH-ire NN probability score. The relative enrichment of SNP-coincident MAPs was nearly two times the enrichment expected by random chance for MAPs above a 0.5 score threshold and decreased to near 1 (no enrichment) at a score threshold of 0.1 (Fig. 8*A*, supplemental Fig. S8*C*).

Within the set of 1732 SNP-coincident MAPs, 338 were distinctly designated as benign or likely benign whereas 667 were designated as pathogenic or likely pathogenic. When comparing the enrichment of SNP-coincident hotspots (>0.196 probability score) relative random expectation, we found that pathogenic hotspots increased proportionally with increasing probability score, whereas benign hotspot enrichment decreased with increasing score threshold (Fig. 8*B*, supplemental Fig. S8*D*). Therefore, the observation of pathogenic SNP-coincident MAPs is greater than expected by random chance and increases with SAPH-ire NN probability score.

We sought to distinguish cases where pathogenic substitution was observed at a PTM site in the human protein (Type-1 coincidence) from cases where the substitution was not a PTM site for the human protein but was nevertheless located within a MAP corresponding to a function potential hotspot (Type-2 coincidence). In Type-1 coincidence there is direct experimental evidence of modification that may be disrupted by the disease mutation. In Type-2 coincidence, evidence for modification of the normal human site that is mutated in disease has not yet been observed but the site is predicted to be important based on evidence from other protein family members. We found that Type-2 hotspot coincidence was 3-fold more abundant than Type-1 coincidence (Fig. 8*C*, 8*D*). In many cases, the SNP-coincident hotspots exhibit known function for the PTM, which may in some cases contribute to the mechanism of disease. We also found 31 hotspots of truly unknown function (U4) that correspond to pathogenic disease-linked mutations in a wide variety of human proteins (supplemental data). In most cases (22/31), the hotspots are Type-2, which means they would go undetected if only the specific human mutated protein were analyzed for coincident PTM. Taken together, these data suggest that considerable improvement in relating disease-specific mutations to protein structure/function can be achieved through the use of function potential hotspot prioritization methods such as SAPH-ire NN.

## DISCUSSION

The need for computational tools intended to convert big data into meaningful information has never been more essential. Post-translational modifications represent one of many forms of emerging big data sets for which this need has remained largely unfulfilled despite an exponential increase in data. We have attempted to address this issue for the subset of PTMs that can be projected onto 3-dimensional protein structures using SAPH-ire (Structural Analysis of PTM Hotspots) and neural network modeling. We have demonstrated that the SAPH-ire NN model is strongly predictive for modified alignment positions that have well documented evidence of biological function, significantly outperforming other previously demonstrated function potential predictors. Because of the universality of the predictive features used in the SAPH-ire NN model, MAPs lacking any evidence of biological function are also subject to analysis, scoring, and therefore rank ordering with respect to all other PTMs. Consequently, unknown function MAPs that harbor feature characteristics similar to known function MAPs become readily apparent and represent putative regulatory elements that can be experimentally targeted to better understand cell behavior and disease in the context of protein mechanism and disregulation.

### 

#### 

##### SAPH-ire NN: Distinction from Other PTM Hotspot Models

Several aspects distinguish SAPH-ire NN from other previously described methods. First, SAPH-ire NN, unlike other methods of hotspot characterization, does not define hotspots based on the identification of nonrandom clusters of PTM along the length of a protein sequence. Although nonrandom PTM clusters do reveal regions of higher PTM density in a domain or domain family, they will in many cases not be associated with functionality. Indeed, we have shown that many if not most functional PTMs do not have neighboring PTMs and that nearest neighbor features are not strongly predictive for functionality (Fig. 4, 5 and supplemental Fig. S4). Second, SAPH-ire NN retains hotspot integrity for single alignment positions rather than clustering PTM data within a ± 2-residue window. As a result, SAPH-ire predictions are based on a precise structural location with well-defined solvent accessible surface area (SASA) for each hotspot, which can be further compared independently between hotspots. Importantly, SAPH-ire NN does not lose the context provided by neighboring alignment positions, but rather, retains this data by incorporating nearest neighbor features into the neural network model. Third, SAPH-ire NN produces a single score for each hotspot, which enables direct comparison of individual hotspots within or between families. In contrast, strategies that employ a residue range window are confined by sequence context that may be restricted to a specific protein family. This is particularly useful for ranking PTM hotspots within a functional protein complex (*e.g.* the heterotrimeric G protein complex), and also identifying multiple positions of potential combinatorial regulation that may occur on separate proteins in the complex. Fourth, SAPH-ire NN is PTM type-agnostic. Rather than focusing on the features of a single PTM type such as phosphorylation, SAPH-ire is anchored by protein structure/function relationships and presumes that the same structure in two different proteins may be important as a modification target (*i.e.* target for different types of PTM within a family) as opposed to a phosphorylation target specifically (for example). Consequently, SAPH-ire provides a means to survey cases in which multiple different PTM types are observed on similar structures within a single protein family (*i.e.* PTM plasticity), but also enables improved functional prediction for MAPs in which very few of any one type of PTM has been observed. Thus, SAPH-ire NN provides valuable predictive information for lower frequency PTMs, unlike phosphorylation or ubiquitination. In summary, we submit that the benefits of a PTM type-agnostic and quantitative prediction method that places PTMs in the context of protein structure without also enforcing artificial clustering of PTMs across multiple alignment positions (*e.g.* ±2 residue windows) provides several advantages for PTM function potential analysis.

##### Possible Improvements to SAPH-ire NN Through the Incorporation of Additional Features

We have shown that the incorporation of six distinct MAP features in the SAPH-ire NN model results in better predictive outcomes than are observed for any feature alone. Thus, it is likely that incorporation of additional MAP features should further improve the SAPH-ire NN model. At present, SAPH-ire does not incorporate PTM dynamics or stoichiometry data—features that have been shown to be predictive for biological function previously (5). Although the availability of these features is increasing, they are less available from proteomics studies conducted to date, due in part to the cost and expertise needed to acquire the information. Availability of modification stoichiometry is even more rare than modification dynamics, and also possibly not as valuable. For example, whereas phosphorylation stoichiometry is critical for the cell cycle-coordinated degradation of the CDK inhibitor, Sic1 (human p27Kip1) (43), it is not critical that all GPCRs on the surface of a cell are phosphorylated to promote desensitization in response to agonist stimulation of a single receptor—though both Sic1 and GPCR phosphorylation mechanisms are essential for normal cell function (44). Indeed, localization-dependent, sub-stoichiometric PTM plays a critical role in GPCR-mediated chemotaxis (45, 46). SAPH-ire NN also does not utilize enzyme/motif matching to estimate PTM function potential. Although these types of relationships have been shown to be predictive for biological function, especially for kinases, they are largely based on predictive rather than experimental evidence; and what experimental data does exist is often limited to a subset of protein families and PTM types.

Beyond the six MAP features described here, we have tested several other MAP and family-descriptive features that did not significantly improve the SAPH-ire NN model (data not shown). Included among these were family member count and intrinsic disorder, which can be widely different between protein families in the SAPH-ire data set. Neither feature alone is strongly predictive for function potential. We have also found that addition or exclusion of different features have unique effects on the distributions of MAPs with different known function source count—some that are worse than others. Thus, detailed analysis beyond ROC-AUC metrics are essential when evaluating the performance of these models. We show here that high-source count, known function MAPs are highly ranked by SAPH-ire NN - independent of family member count (supplemental Fig. S4). During the review of this manuscript, we discovered that an alternative modeling approach was taken in which some (but not all) similar features were used to generate predictive models for phosphorylation (47). Although this suggests that there are indeed more features to be considered, neither the distribution of known function source count nor the contributions of individual features were fully analyzed. Thus, although additional MAP features may be useful, further work will be needed to interrogate their effects on individual MAPs.

##### Exploiting SAPH-ire NN for Understanding the Fundamental Nature of Post-translational Modifications and Genetic Disease

The SAPH-ire NN model was built simply to predict the likelihood that any given MAP is biologically impactful based on characteristics derived from the sequence and structure of PTMs previously determined to have measureable impact on some biological process. Naturally, PTMs that are easily detected (*e.g.* phosphorylation, ubiquitination, acetylation), dominate the empirically derived PTM landscape, which may or may not accurately reflect the natural landscape in living cells - thereby affecting the outcome of the model. The model is also subject to any error inherent in the collection of data as it may occur through PTM detection or through published experimental demonstrations of PTM function. In this as well as our previous study, we rigorously attempt to exclude, correct, or recalibrate data that is nonbiological or resulting as a consequence of arbitrary data curation methods (*e.g.* calibration of sequence alignment between different data formats such as the PDB, UniProt, and other data sources). Indeed, we have found that most data sources are not provided in a manner that enables instantaneous incorporation into SAPH-ire. Having taken these precautions, and further validating SAPH-ire NN output through the analysis of known-function PTMs (Figs. 5–8), we suppose that the final dataset shown here not only provides a list of candidate PTMs likely to impact protein and cellular function, but also informs our knowledge of the nature of post-translational modification in relation to protein structure/function. Restated, the SAPH-ire data set can be used as a foundation for further studies into the process of PTM in general. With this in mind, cutoffs for identification of PTM hotspots, whether based on enrichment over random models or on frequency distributions of known-function source counts, should not be treated as hard-set thresholds. Rather, we would argue that they provide quantitative distinction between different classes of PTM that can be used to better understand their biological nature. Indeed, based on our analysis, the vast majority of PTMs do not exhibit high function potential (Fig. 5). This observation may or may not be a reflection of the source data—including the quality of sample preparation before proteomics mass spectrometry, the quality of PTM identification and localization, as well as the quality of experiments that describe the functionality of PTMs. We have shown that SAPH-ire NN is a strong predictor of MAP function potential despite the absence of explicit PTM data filters. Thus, although improved data quality will surely improve prediction strength, the inclusion of several MAP features into SAPH-ire NN likely also enhance the robustness of such models.

Given the results from this work, we conclude that neural networks and machine learning can provide robust models through which the complexity of PTM feature interactions might be gleaned from biological data. In addition, further improvements to such models may be achieved by inclusion of disease-linked mutations that associate with MAPs, as we have done here (Fig. 8). Not only might such a strategy improve function potential prediction, but it may also reveal previously unrealized connections between several biological features and disease mechanism. We have found several cases in which MAPs can be linked with disease-associated mutations (Fig. 7–8). Interestingly, most cases of coincidence were found as a result of linking PTM and mutation datasets through family alignment position, as opposed to Uniprot ID and native position that is only specific for the protein alone. Thus, biological knowledge from a broad diversity of eukaryotes such as plants, yeast, mice, and humans is used to inform our understanding of human disease. Expanding on these benefits will benefit from further experimental and computational effort that will surely provide greater insight into the nature, evolution and functional landscape of PTMs.

## Supplementary Material

Supplemental Data

## Footnotes

^1^ The abbreviations used are:

PTM: Post-Translational Modification
MAP: Modified Alignment Position, defined by multiple sequence alignment for protein; families used by SAPH-ire.
FP: Function Potential
NN: Neural Network
SAPH-ire: Structural Analysis of PTM Hotspots
ROC: Receiver Operating Characteristic
AUC: Area Under Curve
PRC: PTM Residue Conservation (PTM Res Con); residue conservation in multiple; sequence alignment that can be modified by a specific PTM in question.
PPI: Protein-Protein Interaction or Interface
NC: Neighbor Count; number of neighboring MAPs within a +/- 2-residue window for any given MAP.
NKC: Neighbor Known Count; number of neighboring MAPs that have a known biological function within a +/- 2-residue window for any given MAP.
SASA: Solvent Accessible Surface Area.
