# Supplementary material for: Proteome-wide Structural Analysis of PTM Hotspots Reveals Regulatory Elements Predicted to Impact Biological Function and Disease
Source: Mol Cell Proteomics. 2016 Oct 3;15(11):3513–28. doi: 10.1074/mcp.M116.062331 (PMC5098047; doi:10.1074/mcp.M116.062331)
Supplement: Supplemental Data [file supp_15_11_3513__index.html]

Proteome-Wide Structural Analysis of PTM Hotspots Reveals Regulatory Elements Predicted to Impact Biological Function and Disease — Proteome-wide Structural Analysis of PTM Hotspots Reveals Regulatory Elements Predicted to Impact Biological Function and Disease — Predicting the Biological Impact of PTMs — Supplemental Data 

# Proteome-wide Structural Analysis of PTM Hotspots Reveals Regulatory Elements Predicted to Impact Biological Function and Disease

## Supplemental Data

- Supplemental Data (.pdf, 430 KB) - Supplemental data.
- SAPH-ire MAP Data (.xlsx, 1.9 MB) - Tables showing SAPH-ire MAPs including function potential scores and MAP features.
- KEGG/CLINVAR Analysis of SAPH-ire Data (.xlsx, 12.2 MB) - Contains tables that relate SAPH-ire MAPs to human KEGG pathways and ClinVar disease-linked SNPs.
