# Supplementary material for: Proteome-wide Structural Analysis of PTM Hotspots Reveals Regulatory Elements Predicted to Impact Biological Function and Disease
Source: Mol Cell Proteomics. 2016 Oct 3;15(11):3513–28. doi: 10.1074/mcp.M116.062331 (PMC5098047; doi:10.1074/mcp.M116.062331)
Supplement: Supplemental Data [file 10.1074_M116.062331_mcp.M116.062331-1.pdf]

Supplementary Data

Proteome-Wide Structural Analysis of PTM Hotspots Reveals Regulatory Elements Predicted to Impact Biological Function and Disease

Running Title: Predicting the biological impact of PTMs

Matthew P. Torres*, Henry Dewhurst, Niveda Sundararaman

SUPPLEMENTARY INFORMATION (This PDF File)

Fig. S1. Distribution of disorder tendency relative to number and type of PTM coincidence in MAPs. (A) Frequency of disorder tendency MAPs above (predicted disordered) and below (predicted ordered) 0.5. Frequency is shown for entire SAPH-ire dataset (Total) as well as MAPs with 2-5 coincident PTM types. (B) Same as in A, but with respect to PTM type coincidence. Acetyl/Ubiq coincident PTMs is shown for relative perspective to atypical coincident PTMs indicated.

Fig. S2. ROC-AUC and Chi-Squared hypothesis testing model comparisons. (A) Neural network diagram used here. (B) ROC curves for multiple independent-feature nominal logistic regression and multi-feature neural network model tests. (C) Quantified data from the model comparison shown in B. (D) Each model was tested by pairwise comparison to all other models shown in B and C. ChiSquare hypothesis tests for significance were conducted in each case and shown here. (RD) Rationally Derived model for PTM hotspot function potential.

Fig. S3. Frequency distribution of SAPH-ire NN probability scores for MAPs with 11+ known function source count. The cutoff used to identify function potential hotspots was taken as the score above which 90% of these MAPs were included (0.196).

Fig. S4. (A) Comparison of observed/expected ratio for MAPs with different known-function source count and (B) distribution of known-function source count MAPs (KFSC 11+) with respect to PPI, PTM count, and Family Member Count.

Fig. S5. Diagram of MAP categories described. (A) Frequency distribution of MAPs in the SAPH-ire dataset showing the threshold cutoff for function-potential hotspots (>0.196) and the cutoff used for KEGG pathway mapping studies (>0.35). (B) SAPH-ire NN model was generated based only on the top-most bifurcation (hotspots with either known function or unknown function). High-ranking unknown function hotspots were then categorized further.

Fig. S6. Distribution of hotspots matching specific human Kegg-pathways. (A) Of the 267 truly unknown-function hotspots described in Fig. 6, each PTM contained therein (UID-NP) was related to all available UID-NPs in the database of human pathways via KEGG, resulting in 165 hotspot matches to 210 distinct pathways and 211 distinct human proteins. (B) Scatterplot showing the wide variety of hotspots >35%, relative to family (x-axis). Importantly – even those that do not match are also human PTMs. Thus, the result shows that several high-ranking hotspots, which are predicted to be biologically functional cannot be linked to a distinct pathway based on KEGG analysis.

Fig. S7. Fully expanded scatterplot of all 210 KEGG pathways with function potential hotspots of unknown function (U4). KEGG pathways with UIDs contained with high-ranking (>35% score) hotspots were arranged by maximum and mean hotspot probabilities (as described in Fig. 8). Each pathway is labeled in this graph.

Fig. S8. Tables of observed and expected frequencies. (A) MAP abundance relative to disease-linkage and SAPH-ire NN probability score. (B) Observed/expected ratios for MAPs with varying known-function source count (KFSC). (C) Observed/expected ratios for SNP-coincident MAPs above different probability score thresholds (t). (D) Observed/expected ratios for pathogenic versus benign SNP-coincident MAPs above different scoring thresholds.

ADDITIONAL SUPPLEMENTARY FILES (Files attached independently)

Supplementary File 1 (.xlsx). The SAPH-ire dataset and modeling results. (Tab 1) Key. (Tab 2) SAPH-ire dataset of 31,747 MAPs. (Tab 3) Set of MAPs from Tab 2 that matched to human KEGG pathway (>35% probability score). (Tab 4) Function potential hotspots (MAPs ≥19.6%) that coincide with disease-linked mutations from the ClinVar database.

Fig. S1

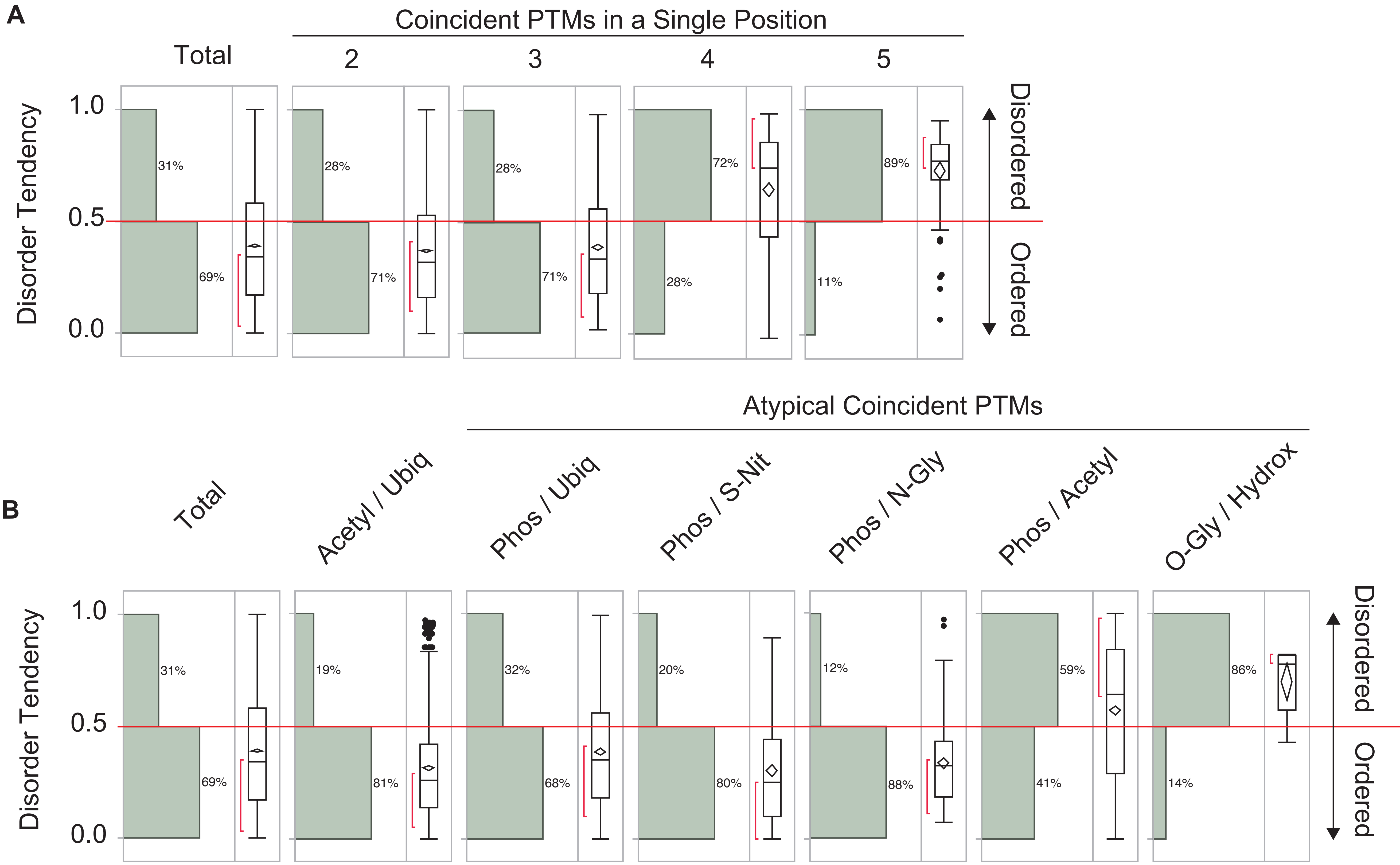
A
Coincident PTMs in a Single Position
Total
2
3
4
5
Disorder Tendency
1.0
0.5
0.0
31%
69%
28%
71%
28%
71%
72%
28%
89%
11%
Disordered
Ordered
B
Atypical Coincident PTMs
Total
Acetyl / Ubiq
Phos / Ubiq
Phos / S-Nit
Phos / N-Gly
Phos / Acetyl
O-Gly / Hydrox
Disorder Tendency
1.0
0.5
0.0
31%
69%
19%
81%
32%
68%
20%
80%
12%
88%
59%
41%
86%
14%
Disordered
Ordered

Fig. S2

A

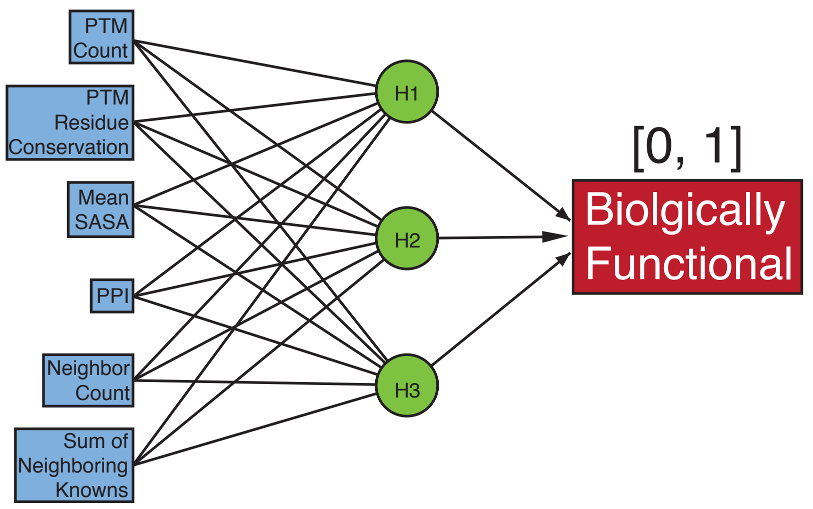

B

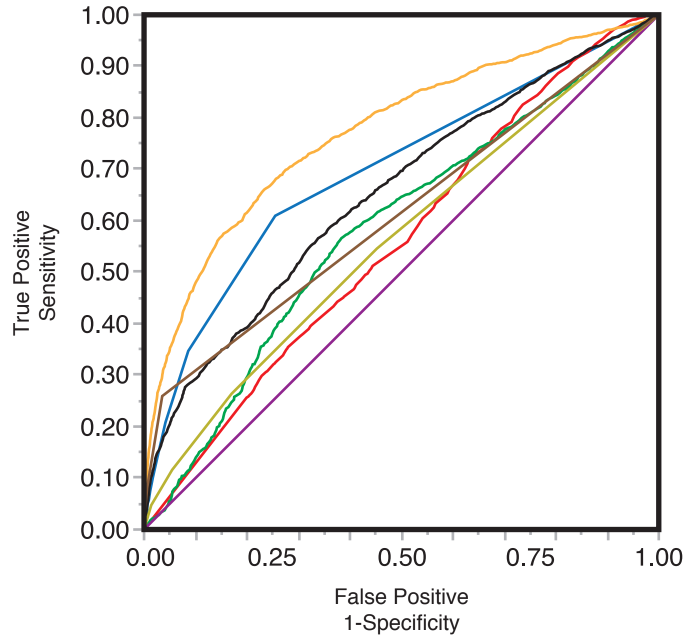

C

Predictor	Generalized r^2	RMSE	N	AUC	Std Error	CI Lower 95%	CI Upper 95%
PTM Count (PC)	0.0315	0.2423	31747	0.6974	0.0060	0.6855	0.7091
PTM Res. Con. (PRC)	0.0061	0.2433	31747	0.5585	0.0063	0.5461	0.5708
Solvent Acc. Surf. Area (SASA)	0.0167	0.2427	31747	0.5859	0.0067	0.5728	0.5989
Protein-protein Interface (PPI)	-0.0001	0.2435	31747	0.5009	0.0051	0.4899	0.5100
Neural Network model (NN)	0.1497	0.2308	31747	0.7744	0.0060	0.7625	0.7859
Rationally-Derived model (RD)	0.0489	0.2404	31747	0.6532	0.0067	0.6399	0.6662
Neighbor Count (NC)	0.0126	0.2428	31747	0.5631	0.0064	0.5505	0.5756
Neighboring Known Count (NKC)	0.0890	0.2360	31747	0.6126	0.0049	0.6029	0.6223

D

Predictor	vs. Predictor	AUC Comparisons: Difference	Std Error	Lower 95%	Upper 95%	ChiSquare	Prob>ChiSq
PC	PRC	0.1390	0.0084	0.1226	0.1554	274.53	p<<<0.0001
PC	SASA	0.1116	0.0085	0.0948	0.1283	171.15	p<<<0.0001
PC	PPI	0.1975	0.0078	0.1823	0.2127	645.34	p<<<0.0001
PC	NN	-0.077	0.0052	-0.087	-0.067	218.97	p<<<0.0001
PC	RD	0.0442	0.0066	0.0314	0.0571	45.288	p<<<0.0001
PC	NC	0.1344	0.0077	0.1193	0.1494	306.95	p<<<0.0001
PC	NKC	0.0848	0.0074	0.0702	0.0993	130.48	p<<<0.0001
PRC	SASA	-0.027	0.0094	-0.046	-0.009	8.6145	p=0.0033
PRC	PPI	0.0585	0.0084	0.0420	0.0750	48.075	p<<<0.0001
PRC	NN	-0.216	0.0071	-0.230	-0.202	933.7	p<<<0.0001
PRC	RD	-0.095	0.0069	-0.108	-0.081	190.2	p<<<0.0001
PRC	NC	-0.005	0.0101	-0.024	0.0152	0.2105	n.s.
PRC	NKC	-0.054	0.0085	-0.071	-0.037	40.33	p<<<0.0001
SASA	PPI	0.0859	0.0093	0.0677	0.1041	85.663	p<<<0.0001
SASA	NN	-0.189	0.0076	-0.204	-0.174	607.3	p<<<0.0001
SASA	RD	-0.067	0.0053	-0.078	-0.057	159.3	p<<<0.0001
SASA	NC	0.0228	0.0087	0.0058	0.0398	6.8757	p=0.0087
SASA	NKC	-0.027	0.0078	-0.042	-0.011	11.643	p<<<0.0001
PPI	NN	-0.274	0.0081	-0.290	-0.258	1134.2	p<<<0.0001
PPI	RD	-0.153	0.0086	-0.170	-0.136	314.93	p<<<0.0001
PPI	NC	-0.063	0.0080	-0.079	-0.047	62.2	p<<<0.0001
PPI	NKC	-0.113	0.0070	-0.126	-0.099	256.96	p<<<0.0001
NN	RD	0.1212	0.0059	0.1096	0.1328	417.89	p<<<0.0001
NN	NC	0.2113	0.0085	0.1947	0.2279	622.25	p<<<0.0001
NN	NKC	0.1618	0.0059	0.1502	0.1733	751.76	p<<<0.0001
RD	NC	0.0901	0.0090	0.0726	0.1077	101.19	p<<<0.0001
RD	NKC	0.0406	0.0081	0.0247	0.0564	25.095	p<<<0.0001
NC	NKC	-0.050	0.0054	-0.060	-0.039	83.609	p<<<0.0001

Test	ChiSquare	DF	Prob>ChiSq
All AUCs equal	1828.53	7	p<<<0.0001

Fig. S3

Distribution of High Source Count MAPs (11+ sources)

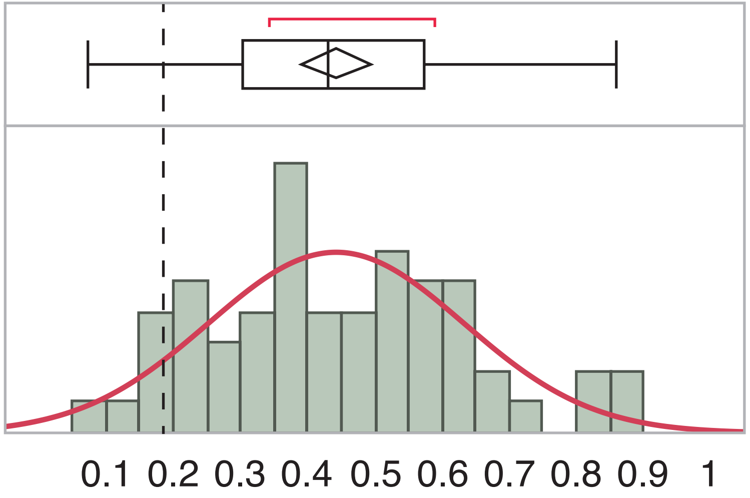

Quantiles

100.0%	maximum	0.8609740641
99.5%		0.8609740641
97.5%		0.8566707296
90.0%		0.6768364812
75.0%	quartile	0.5717404408
50.0%	median	0.4292616468
25.0%	quartile	0.3017795331
**10.0%**		**0.1960635817**
2.5%		0.0947106267
0.5%		0.0721029591
0.0%	minimum	0.0721029591

Summary Statistics

Mean	0.4424575
Std Dev	0.1900793
Std Err Mean	0.0249586
Upper 95% Mean	0.4924363
Lower 95% Mean	0.3924787
N	58

Fig. S4

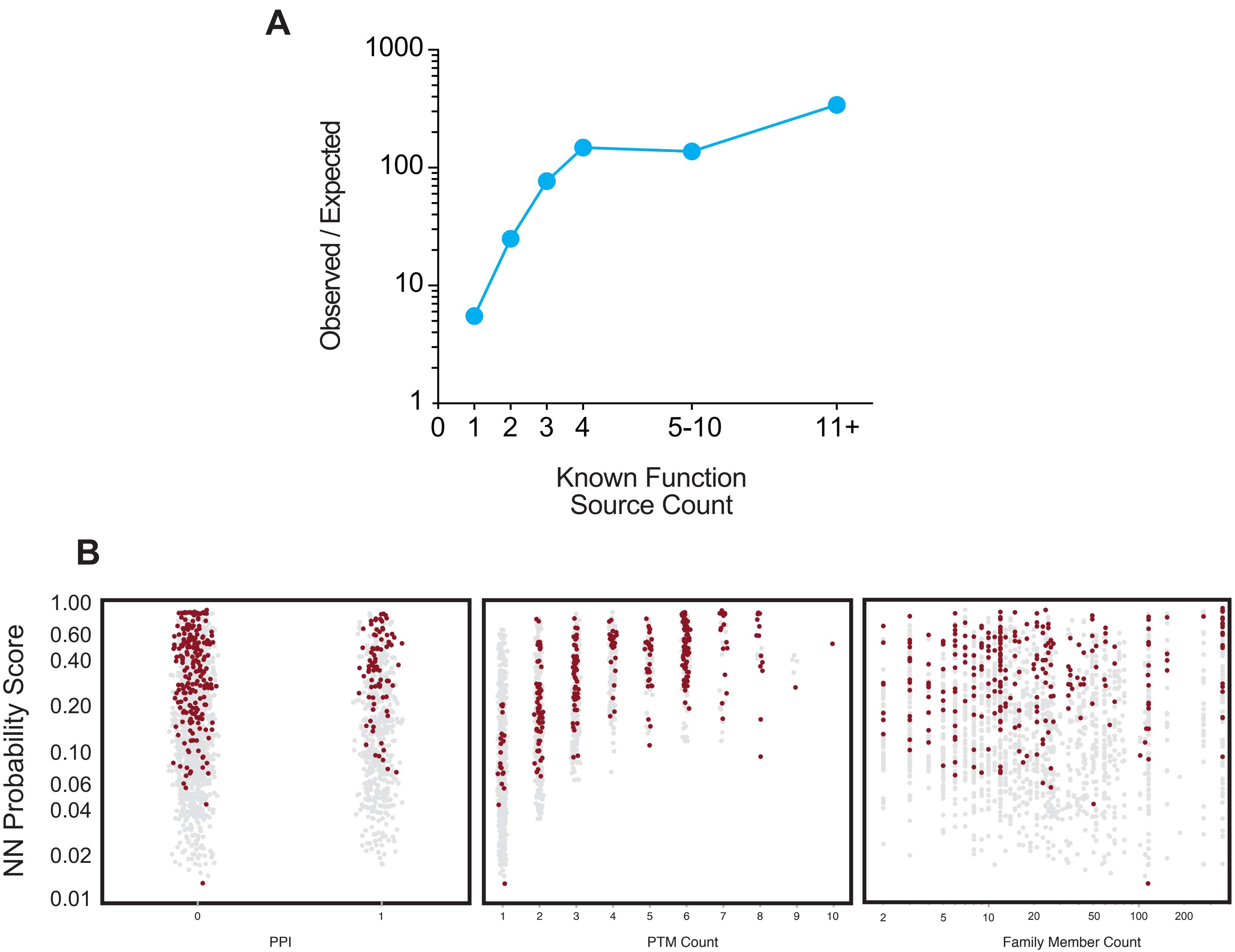
A
1000
100
10
1
Observed / Expected
0
1
2
3
4
5-10
11+
Known Function
Source Count
B
NN Probability Score
1.00
0.60
0.40
0.20
0.10
0.06
0.04
0.02
0.01
0
1
PPI
1
2
3
4
5
6
7
8
9
10
PTM Count
2
5
10
20
50
100
200
Family Member Count

Fig. S5

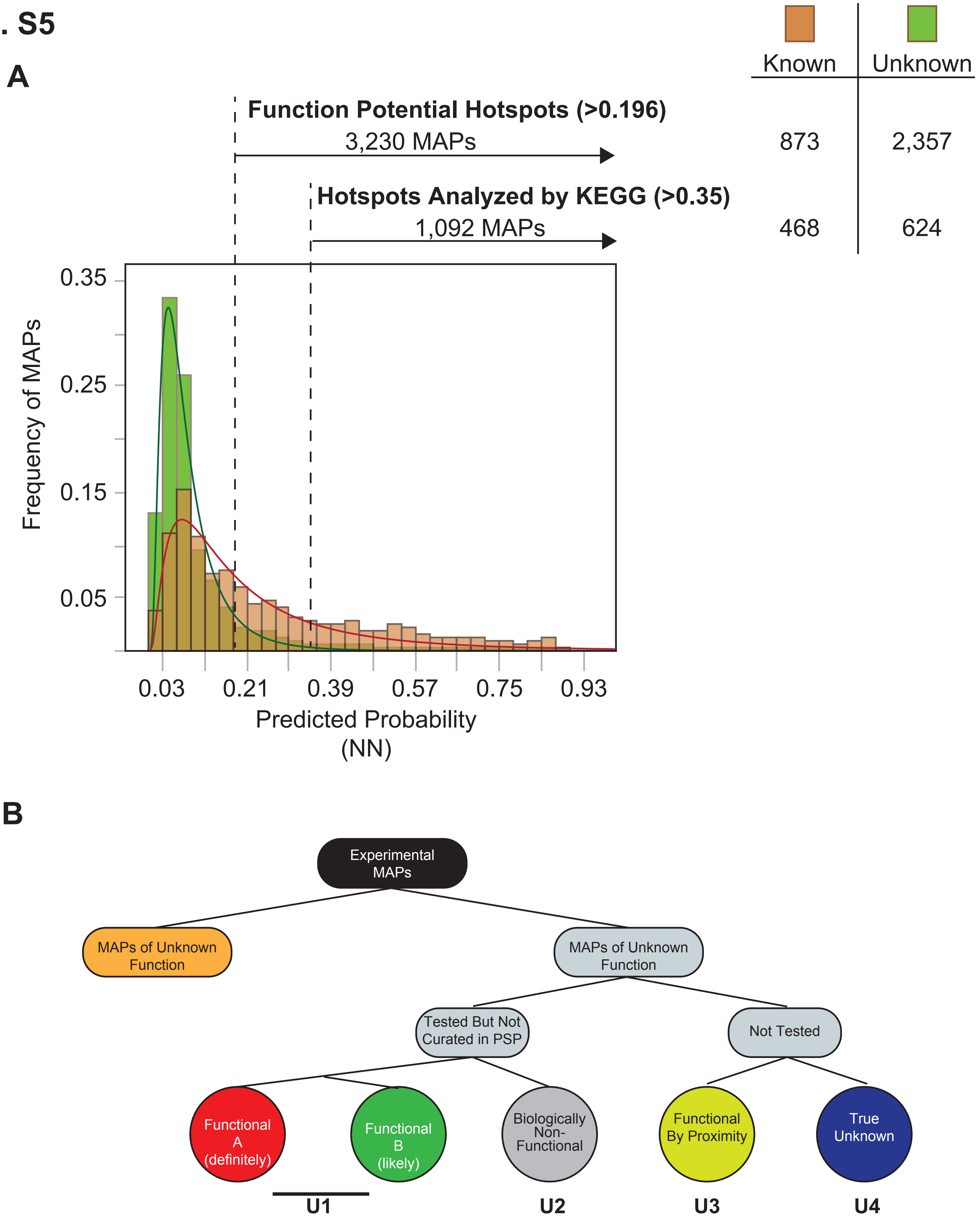
A
Known
Unknown
Function Potential Hotspots (>0.196)
3,230 MAPs
873
2,357
Hotspots Analyzed by KEGG (>0.35)
1,092 MAPs
468
624
Frequency of MAPs
0.35
0.25
0.15
0.05
0.03
0.21
0.39
0.57
0.75
0.93
Predicted Probability
(NN)
B
Experimental MAPs
MAPs of Unknown Function
MAPs of Unknown Function
Tested But Not Curated in PSP
Not Tested
Functional A (definitely)
Functional B (likely)
Biologically Non-Functional
Functional By Proximity
True Unknown
U1
U2
U3
U4

Fig. S6

A

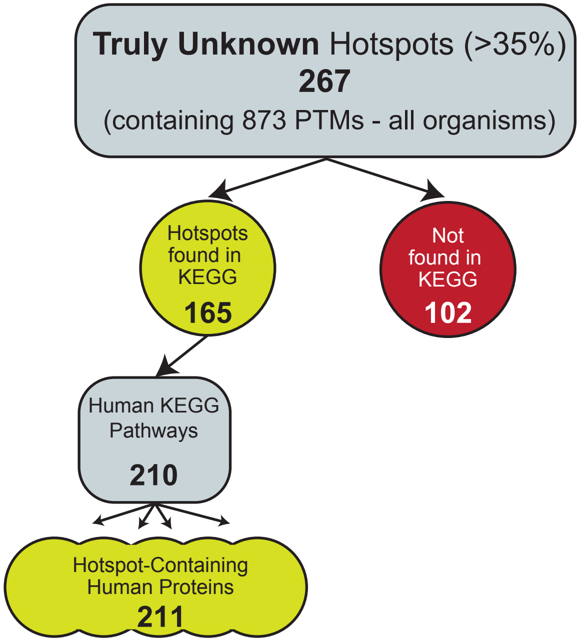

Truly Unknown Hotspots (>35%)
267
(containing 873 PTMs - all organisms)
Hotspots found in KEGG
165
Not found in KEGG
102
Human KEGG Pathways
210
Hotspot-Containing Human Proteins
211

B

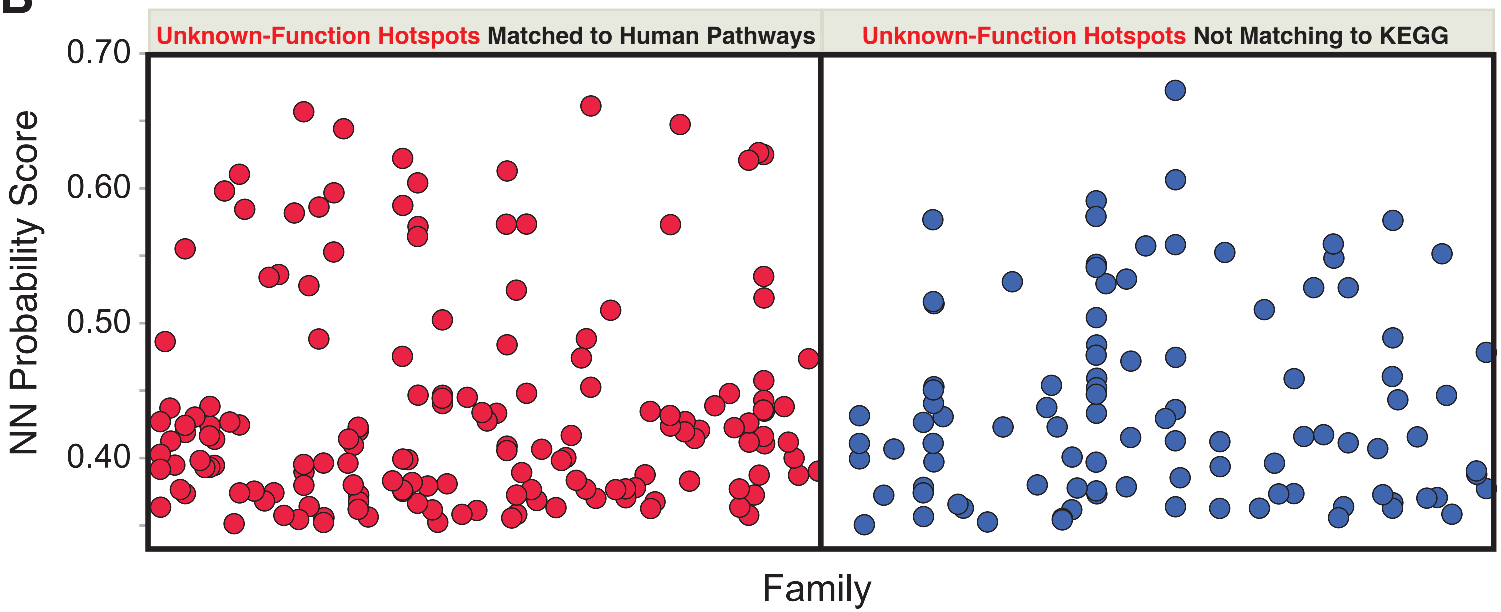

Unknown-Function Hotspots Matched to Human Pathways
Unknown-Function Hotspots Not Matching to KEGG
0.70
0.60
0.50
0.40
NN Probability Score
Family

Fig. S7

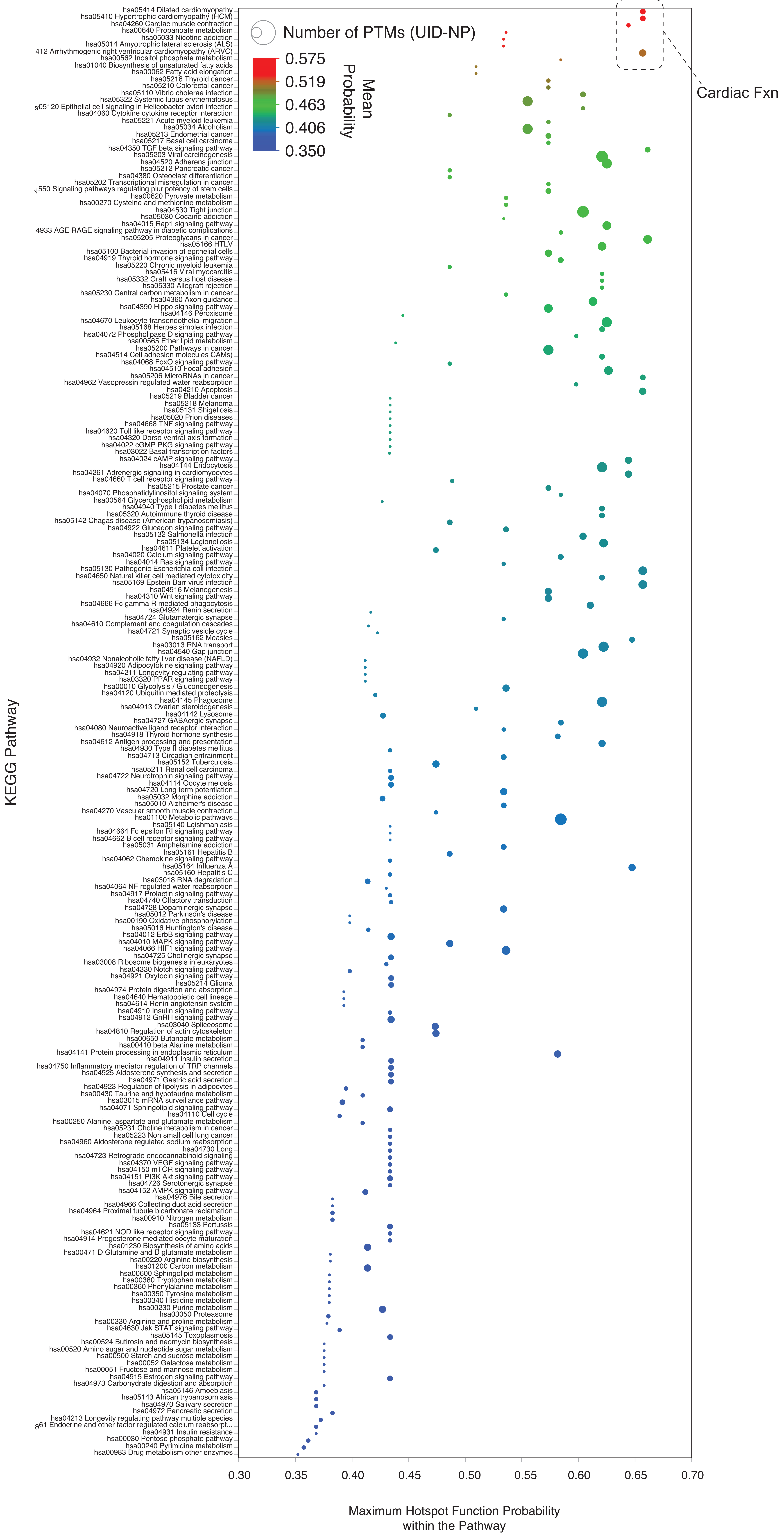

Number of PTMs (UID-NP)
0.575
0.519
0.463
0.406
0.350
Mean Probability
Cardiac Fxn
KEGG Pathway
hsa05414 Dilated cardiomyopathy
hsa05410 Hypertrophic cardiomyopathy (HCM)
hsa04260 Cardiac muscle contraction
hsa00640 Propanoate metabolism
hsa05033 Nicotine addiction
hsa05014 Amyotrophic lateral sclerosis (ALS)
412 Arrhythmogenic right ventricular cardiomyopathy (ARVC)
hsa00562 Inositol phosphate metabolism
hsa01040 Biosynthesis of unsaturated fatty acids
hsa00062 Fatty acid elongation
hsa05216 Thyroid cancer
hsa05210 Colorectal cancer
hsa05110 Vibrio cholerae infection
hsa05322 Systemic lupus erythematosus
a05120 Epithelial cell signaling in Helicobacter pylori infection
hsa04060 Cytokine cytokine receptor interaction
hsa05221 Acute myeloid leukemia
hsa05034 Alcoholism
hsa05213 Endometrial cancer
hsa05217 Basal cell carcinoma
hsa04350 TGF beta signaling pathway
hsa05203 Viral carcinogenesis
hsa04520 Adherens junction
hsa05212 Pancreatic cancer
hsa04380 Osteoclast differentiation
hsa05202 Transcriptional misregulation in cancer
4550 Signaling pathways regulating pluripotency of stem cells
hsa00620 Pyruvate metabolism
hsa00270 Cysteine and methionine metabolism
hsa04530 Tight junction
hsa05030 Cocaine addiction
hsa04015 Rap1 signaling pathway
4933 AGE RAGE signaling pathway in diabetic complications
hsa05205 Proteoglycans in cancer
hsa05166 HTLV
hsa05100 Bacterial invasion of epithelial cells
hsa04919 Thyroid hormone signaling pathway
hsa05220 Chronic myeloid leukemia
hsa05416 Viral myocarditis
hsa05332 Graft versus host disease
hsa05330 Allograft rejection
hsa05230 Central carbon metabolism in cancer
hsa04360 Axon guidance
hsa04390 Hippo signaling pathway
hsa04146 Peroxisome
hsa04670 Leukocyte transendothelial migration
hsa05168 Herpes simplex infection
hsa04072 Phospholipase D signaling pathway
hsa00565 Ether lipid metabolism
hsa05200 Pathways in cancer
hsa04514 Cell adhesion molecules CAMs)
hsa04068 FoxO signaling pathway
hsa04510 Focal adhesion
hsa05206 MicroRNAs in cancer
hsa04962 Vasopressin regulated water reabsorption
hsa04210 Apoptosis
hsa05219 Bladder cancer
hsa05218 Melanoma
hsa05131 Shigellosis
hsa05020 Prion diseases
hsa04668 TNF signaling pathway
hsa04620 Toll like receptor signaling pathway
hsa04320 Dorso ventral axis formation
hsa04022 cGMP PKG signaling pathway
hsa03022 Basal transcription factors
hsa04024 cAMP signaling pathway
hsa04144 Endocytosis
hsa04261 Adrenergic signaling in cardiomyocytes
hsa04660 T cell receptor signaling pathway
hsa05215 Prostate cancer
hsa04070 Phosphatidylinositol signaling system
hsa00564 Glycerophospholipid metabolism
hsa04940 Type I diabetes mellitus
hsa05320 Autoimmune thyroid disease
hsa05142 Chagas disease (American trypanosomiasis)
hsa04922 Glucagon signaling pathway
hsa05132 Salmonella infection
hsa05134 Legionellosis
hsa04611 Platelet activation
hsa04020 Calcium signaling pathway
hsa04014 Ras signaling pathway
hsa05130 Pathogenic Escherichia coli infection
hsa04650 Natural killer cell mediated cytotoxicity
hsa05169 Epstein Barr virus infection
hsa04916 Melanogenesis
hsa04310 Wnt signaling pathway
hsa04666 Fc gamma R mediated phagocytosis
hsa04924 Renin secretion
hsa04724 Glutamatergic synapse
hsa04610 Complement and coagulation cascades
hsa04721 Synaptic vesicle cycle
hsa05162 Measles
hsa03013 RNA transport
hsa04540 Gap junction
hsa04932 Nonalcoholic fatty liver disease (NAFLD)
hsa04920 Adipocytokine signaling pathway
hsa04211 Longevity regulating pathway
hsa03320 PPAR signaling pathway
hsa00010 Glycolysis / Gluconeogenesis
hsa04120 Ubiquitin mediated proteolysis
hsa04145 Phagosome
hsa04913 Ovarian steroidogenesis
hsa04142 Lysosome
hsa04727 GABAergic synapse
hsa04080 Neuroactive ligand receptor interaction
hsa04918 Thyroid hormone synthesis
hsa04612 Antigen processing and presentation
hsa04930 Type II diabetes mellitus
hsa04713 Circadian entrainment
hsa05152 Tuberculosis
hsa05211 Renal cell carcinoma
hsa04722 Neurotrophin signaling pathway
hsa04114 Oocyte meiosis
hsa04720 Long term potentiation
hsa05032 Morphine addiction
hsa05010 Alzheimer's disease
hsa04270 Vascular smooth muscle contraction
hsa01100 Metabolic pathways
hsa05140 Leishmaniasis
hsa04664 Fc epsilon RI signaling pathway
hsa04662 B cell receptor signaling pathway
hsa05031 Amphetamine addiction
hsa05161 Hepatitis B
hsa04062 Chemokine signaling pathway
hsa05164 Influenza A
hsa05160 Hepatitis C
hsa03018 RNA degradation
hsa04064 NF regulated water reabsorption
hsa04917 Prolactin signaling pathway
hsa04740 Olfactory transduction
hsa04728 Dopaminergic synapse
hsa05012 Parkinson's disease
hsa00190 Oxidative phosphorylation
hsa05016 Huntington's disease
hsa04012 ErbB signaling pathway
hsa04010 MAPK signaling pathway
hsa04066 HIF1 signaling pathway
hsa04725 Cholinergic synapse
hsa03008 Ribosome biogenesis in eukaryotes
hsa04330 Notch signaling pathway
hsa04921 Oxytocin signaling pathway
hsa05214 Glioma
hsa04974 Protein digestion and absorption
hsa04640 Hematopoietic cell lineage
hsa04614 Renin angiotensin system
hsa04910 Insulin signaling pathway
hsa04912 GnRH signaling pathway
hsa03040 Spliceosome
hsa04810 Regulation of actin cytoskeleton
hsa00650 Butanoate metabolism
hsa00410 beta Alanine metabolism
hsa04141 Protein processing in endoplasmic reticulum
hsa04911 Insulin secretion
hsa04750 Inflammatory mediator regulation of TRP channels
hsa04925 Aldosterone synthesis and secretion
hsa04971 Gastric acid secretion
hsa04923 Regulation of lipolysis in adipocytes
hsa00430 Taurine and hypotaurine metabolism
hsa03015 mRNA surveillance pathway
hsa04071 Sphingolipid signaling pathway
hsa04110 Cell cycle
hsa00250 Alanine, aspartate and glutamate metabolism
hsa05231 Choline metabolism in cancer
hsa05223 Non small cell lung cancer
hsa04960 Aldosterone regulated sodium reabsorption
hsa04730 Long
hsa04723 Retrograde endocannabinoid signaling
hsa04370 VEGF signaling pathway
hsa04150 mTOR signaling pathway
hsa04151 PI3K Akt signaling pathway
hsa04726 Serotonergic synapse
hsa04152 AMPK signaling pathway
hsa04976 Bile secretion
hsa04966 Collecting duct acid secretion
hsa04964 Proximal tubule bicarbonate reclamation
hsa00910 Nitrogen metabolism
hsa05133 Pertussis
hsa04621 NOD like receptor signaling pathway
hsa04914 Progesterone mediated oocyte maturation
hsa01230 Biosynthesis of amino acids
hsa00471 D Glutamine and D glutamate metabolism
hsa00220 Arginine biosynthesis
hsa01200 Carbon metabolism
hsa00600 Sphingolipid metabolism
hsa00380 Tryptophan metabolism
hsa00360 Phenylalanine metabolism
hsa00350 Tyrosine metabolism
hsa00340 Histidine metabolism
hsa00230 Purine metabolism
hsa03050 Proteasome
hsa00330 Arginine and proline metabolism
hsa04630 Jak STAT signaling pathway
hsa05145 Toxoplasmosis
hsa00524 Butirosin and neomycin biosynthesis
hsa00520 Amino sugar and nucleotide sugar metabolism
hsa00500 Starch and sucrose metabolism
hsa00052 Galactose metabolism
hsa00051 Fructose and mannose metabolism
hsa04915 Estrogen signaling pathway
hsa04973 Carbohydrate digestion and absorption
hsa05146 Amoebiasis
hsa05143 African trypanosomiasis
hsa04970 Salivary secretion
hsa04972 Pancreatic secretion
hsa04213 Longevity regulating pathway multiple species
961 Endocrine and other factor regulated calcium reabsorpt...
hsa04931 Insulin resistance
hsa00030 Pentose phosphate pathway
hsa00240 Pyrimidine metabolism
hsa00983 Drug metabolism other enzymes
0.30
0.35
0.40
0.45
0.50
0.55
0.60
0.65
0.70
Maximum Hotspot Function Probability
within the Pathway

Fig. S8

A

Category	Count
Total MAPs in the SAPH-ire dataset	31747
Total SNPs from ClinVar	24951
Total SNP-coincident MAPs	1732
Total MAPs above 0.1 cutoff	8504
Total MAPs above 0.196 cutoff	3230
Total MAPs above 0.35 cutoff	1092
Total MAPs above 0.50 cutoff	455
Total MAPs above 0.60 cutoff	215

B

Known Function Source Count Category	1	2	3	4	5-10	11+
Total Known-Function MAPs	2010	2010	2010	2010	2010	2010
Total MAPs at KFSC	1234	345	138	91	144	58
Total MAPs >0.196	3230	3230	3230	3230	3230	3230
KFSC MAPs >0.196	421	150	74	62	113	53
Expected Frequency (Random)	0.062	0.017	0.007	0.005	0.006	0.003
Observed Frequency	0.341	0.435	0.536	0.681	0.785	0.914
Fold Enrichment over random	5.462	24.897	76.766	147.913	137.194	340.619

C

NN Probability Cutoff (t)	0.1	0.196	0.35	0.5	TOTAL
MAPs Observed	8504	3230	1092	455	31747
Observed SNP-Coincident MAPs above t	564	270	109	49	1732
Expected Frequency (MAP >t)	0.268	0.102	0.034	0.014	
Observed Frequency(SNP/MAP >t)	0.326	0.156	0.063	0.028	
Observed/Expected	1.216	1.532	1.830	1.974	

D

ClinVar / SAPH-ire Observed and Expected	Pathogenic	Benign
Total Mutations from ClinVar	24951	24951
Pathogenic or Benign mutations	9289	4547
Expected frequency of mutations >0.196	0.038	0.019
Expected frequency of mutations >0.35	0.013	0.006
Expected frequency of mutations >0.50	0.005	0.003
Expected frequency of mutations >0.60	0.003	0.001
Observed frequency of mutations >0.196	0.056	0.033
Observed frequency of mutations >0.35	0.023	0.010
Observed frequency of mutations >0.50	0.010	0.005
Observed frequency of mutations >0.60	0.006	0.001
Observed/Expected >0.196	1.48	1.77
Observed/Expected >0.35	1.80	1.66
Observed/Expected >0.50	1.84	1.77
Observed/Expected >0.60	2.52	0.47
